# Development of a versatile enrichment analysis tool reveals associations between the maternal brain and mental health disorders, including autism

**DOI:** 10.1186/1471-2202-14-147

**Published:** 2013-11-19

**Authors:** Brian E Eisinger, Michael C Saul, Terri M Driessen, Stephen C Gammie

**Affiliations:** 1 Department of Zoology, University of Wisconsin-Madison, Madison, Wisconsin, USA; 2 Neuroscience Training Program, University of Wisconsin-Madison, Madison, Wisconsin, USA

**Keywords:** Autism, Schizphrenia, Bipolar disorder, Depression, ADHD, Mental health, Enrichment analysis, Maternal brain, Lateral septum

## Abstract

**Background:**

A recent study of lateral septum (LS) suggested a large number of autism-related genes with altered expression in the postpartum state. However, formally testing the findings for enrichment of autism-associated genes proved to be problematic with existing software. Many gene-disease association databases have been curated which are not currently incorporated in popular, full-featured enrichment tools, and the use of custom gene lists in these programs can be difficult to perform and interpret. As a simple alternative, we have developed the Modular Single-set Enrichment Test (MSET), a minimal tool that enables one to easily evaluate expression data for enrichment of any conceivable gene list of interest.

**Results:**

The MSET approach was validated by testing several publicly available expression data sets for expected enrichment in areas of autism, attention deficit hyperactivity disorder (ADHD), and arthritis. Using nine independent, unique autism gene lists extracted from association databases and two recent publications, a striking consensus of enrichment was detected within gene expression changes in LS of postpartum mice. A network of 160 autism-related genes was identified, representing developmental processes such as synaptic plasticity, neuronal morphogenesis, and differentiation. Additionally, maternal LS displayed enrichment for genes associated with bipolar disorder, schizophrenia, ADHD, and depression.

**Conclusions:**

The transition to motherhood includes the most fundamental social bonding event in mammals and features naturally occurring changes in sociability. Some individuals with autism, schizophrenia, or other mental health disorders exhibit impaired social traits. Genes involved in these deficits may also contribute to elevated sociability in the maternal brain. To date, this is the first study to show a significant, quantitative link between the maternal brain and mental health disorders using large scale gene expression data. Thus, the postpartum brain may provide a novel and promising platform for understanding the complex genetics of improved sociability that may have direct relevance for multiple psychiatric illnesses. This study also provides an important new tool that fills a critical analysis gap and makes evaluation of enrichment using any database of interest possible with an emphasis on ease of use and methodological transparency.

## Background

Large scale, genome wide expression studies (such as exon microarrays, promoter microarrays, ChIP-on-CHIPS, and next gen sequencing) have allowed researchers to move from traditional approaches that focus on analyzing one or a few genes to new methods that generate high volumes of simultaneously collected expression data for tens of thousands of genes. This represents a powerful opportunity to identify alterations in large scale biological processes relevant to the study being performed, but it has also created a highly complex data environment with a unique set of challenges. Over the last decade, new software for analyzing enrichment of functionally related gene groups in large scale gene expression data has been developed and implemented successfully. There is an abundance of programs currently available that perform enrichment analyses in a variety of ways, each with its own advantages, disadvantages, and suitable applications [1-6].

Our laboratory recently carried out microarray experiments to explore gene expression changes in the lateral septum (LS) of outbred mice associated with the transition from a virgin to lactating, maternal state. The LS is part of an interconnected network of brain regions known to be critically important in social and maternal behavior [7,8]. Several enrichment analyses were performed, including the Broad Institute’s Gene Set Enrichment Analysis (GSEA) and the NIH’s Database for Annotation, Visualization and Integrated Discovery (DAVID) [1,3,9,10]. These programs proved to be fruitful in profiling large scale alterations in functionally related networks of genes in the postpartum LS, and enrichment was discovered for pathways related to ion channel activity, developmental processes, cyclic nucleotide metabolism, nucleosome components, and the Ras superfamily of small GTPases [11].

We began to notice that numerous genes among the most significant expression results in maternal LS had strong links to autism. This was intriguing because both autism and maternity involve behavioral and emotional alterations. Autism is a pervasive class of spectrum disorders typically characterized by deficits in social interaction, impaired communication abilities, and patterns of repetitive behavior [12,13]. Autistic individuals can have difficulty empathizing with others, developing spoken language, and maintaining relationships [14,15]. In comparison, the transition from a virgin to postpartum state represents a profound transformation in which an animal that was previously concerned mostly with its own survival develops heightened aspects of sociability and becomes critically focused on the wellbeing of its offspring [16]. Because the establishment of the maternal phenotype is a natural process, the postpartum, outbred mouse provides a useful model for exploring the genetics of social behavior and its dysregulation [17]. Oxytocin and vasopressin signaling in rodent maternal care has recently been investigated for its translational relevance to autism spectrum disorders in humans [18-22]. Moving from a single molecule focus to large scale genetic comparisons between the maternal brain and autism may provide broader insight to the neurological basis of sociability and contribute to our understanding of mental health disorders.

We recognized that it would be valuable to formally test for enrichment of autism-related genes in the maternal LS, but we quickly encountered several difficulties. Most available enrichment analysis tools operate solely on functional and pathway association databases, such as Gene Ontology (GO) terms or Kyoto Encyclopedia of Genes and Genomes (KEGG) pathways, and do not include curated groups of disease-linked genes [1]. The handful of programs that do support disease ontology make use of only few predominant disease databases, such as the Online Mendelian Inheritance in Man (OMIM) and the Genetic Association Database (GAD) [23-25]. These databases are detailed and comprehensive, but because enrichment analysis is critically sensitive to the gene lists used as input, it is detrimental to be restricted to a single source of disease associations. There is a vibrant landscape of researchers and organizations that build and maintain numerous genetic disease association databases, but these resources are not linked to any formal means of enrichment analysis (Additional file 1: Table S1) [26-29]. In addition to comprehensive collections of gene-disease associations that encompass many diseases, it is not uncommon for a high profile disorder to engender a community of foundations, initiatives, and projects dedicated to furthering genetic research for that disease alone. For autism in particular, such notable groups include AutismKB by Peking University’s Center for Bioinformatics [30], AutDB by the nonprofit organization Mindspec [31], and the Autism Genetic Database (AGD) [32]. Furthermore, ongoing research continuously generates new sets of candidate autism genes through a variety of novel techniques. For example, one recent publication used a novel Genome-wide Association Study method that reduces statistical noise and minimizes false positives to build a list of autism candidate genes [33], while another group used a functional profile of 84 autism-linked genes to screen the human genome for a larger set of predictive autism susceptibility genes [34].

To make use of this deep and varied field of autism genetics in enrichment analysis, it was clear that researchers must be allowed to create and use custom gene lists in a statistically robust hypothesis testing method. While some popular enrichment packages permit the inclusion of user-generated gene lists, the practicality of this feature often suffers from overly complex, opaque computations and internal significance corrections, a loss of large scale expression data fidelity through forced species-to-species conversions, and a heavy reliance on gene ranking methods and myriad configuration options that can easily lead to vastly different results that may be spurious or open to misinterpretation. As a simple alternative that focuses on the use of custom gene lists, we developed the Modular Single-set Enrichment Test (MSET) – a minimal randomization script that allows researchers to create and utilize any list of disease-associated genes as modules for enrichment testing and gene identification. MSET is designed to be accessible and easy for researchers of any background to use; it runs in R (free to download), requires only a single command line to execute, and subsequently interacts with the user via a simple window and text prompt interface. The lightweight architecture of the program confers independence from platform specific or species specific annotation systems and introduces minimal additional handling of expression data. Such an approach trades the exploratory aspect of full-featured enrichment software that searches for and scores enrichment across numerous biological pathways for a more focused hypothesis testing analysis for enrichment in diseases of interest. This allows investigators to use the most current database for a given disorder, customize their own, or repeat the analysis using numerous databases to generate a comprehensive meta-analysis and obtain high confidence in their results. Another important aspect of MSET is that it quickly highlights genes of interest that can be examined in follow up studies. As a result of its versatility, modular power, and ease of use, MSET represents an improvement over existing tools for performing enrichment analysis with independently assembled disease-associated gene sets.

Using the MSET approach, we discovered a striking consensus of enrichment for expression changes of autism-related genes across nine independent databases in the maternal LS compared to virgin. We found additional evidence of enrichment in the maternal LS for several other mental health diseases, including bipolar disorder, depression, and schizophrenia. This article will serve to present the biological findings from our data, as well as to introduce and demonstrate the novel enrichment tool used to generate them.

## Implementation

MSET is a script written in R that calls upon text files saved within the same folder as the script itself. Outlined simply, the MSET script is a randomization test that calculates the probability of randomly generating a set of microarray results that includes as many disease-associated genes as does a set of results from an actual experiment. It requires two input files: 1) a gene list of interest from a disease association database (autism-associated genes, schizophrenia-associated genes, etc.) for which enrichment will be evaluated, and 2) a full list of all microarray gene results ordered by significance. After choosing a threshold for how many of the top genes will be examined for enrichment, MSET saves them as a list of significant expression results. This list is separate from the full microarray results, which is also known as the microarray background. MSET then generates a specified number of simulated results by sampling randomly and without replacement from the microarray background. It counts the number of genes in each set of simulated results that appear in the gene list of interest (matches to database), and the p-value is calculated as the proportion of simulated results which contained at least as many matches to database as the actual significant expression results.

MSET analysis is performed at the gene level, but microarray platforms can include probes that target intergenic areas and lack a gene ID annotation. Prior to permutation testing, these non-annotated reads are removed from the significant microarray results. Because some genes in microarrays may be assigned multiple probe sets, duplicate gene IDs are also removed from the top selected significant results list, such that each gene is represented only once. The same treatments are applied to the gene list of interest database to ensure completeness and uniqueness. For the microarray background, targets without annotations to genes are removed, but duplicate targets for a single gene are retained. This is part of a strategy to preserve true-to-platform probabilities when sampling targets from the background to build simulated results.

After the user specifies the number of simulated results to be generated from the microarray background (e.g., 10,000), the script begins sampling without replacement. For each simulated set of results, MSET actually builds a list that is twice the length of the true significant results, removes duplicate genes from it, and then discards the excess genes such that the simulated results list is then the same length as the actual results. In this way, genes that are represented by many probes have a greater chance to be included in the simulated results, corresponding to their “weight” in the microarray platform. This allows for simulated results that are true to the particular microarray platform.

MSET output consists of two elements; a graph of the probability distribution of matches to database in simulated results, and a text readout with a variety of information about the input gene lists, the simulated results, and enrichment p-value. It also displays the individual disease-associated genes from the database of interest that appeared in the actual microarray results.

All expression data used in this study were taken from published microarray studies that have been uploaded to NCBI’s Gene Expression Omnibus (GEO, http://www.ncbi.nlm.nih.gov/geo/) or an institutional website. Details of experimental design are provided in the original publications. Postpartum LS expression data were prepared in our laboratory with the Probe Logarithmic Intensity Error (PLIER) algorithm. For data accessed via the GEO, gene expression changes between experimental groups were ranked by the built in GEO2R tool. While a researcher can decide which probe set algorithm is optimal for their particular microarray experiment, differences in the current study are small enough that both methods yield approximately similarly ordered results.

The data sets supporting the results of this article are available via NCBI’s Gene Expression Omnibus (GEO) with the following links and accession numbers.

http://www.ncbi.nlm.nih.gov/geo/query/acc.cgi?acc=GSE27492.htmlhttp://www.ncbi.nlm.nih.gov/geo/query/acc.cgi?acc=GSE43627http://www.ncbi.nlm.nih.gov/geo/query/acc.cgi?acc=GSE22371http://www.ncbi.nlm.nih.gov/geo/query/acc.cgi?acc=GSE33619.

## Results

**Figure 1:**
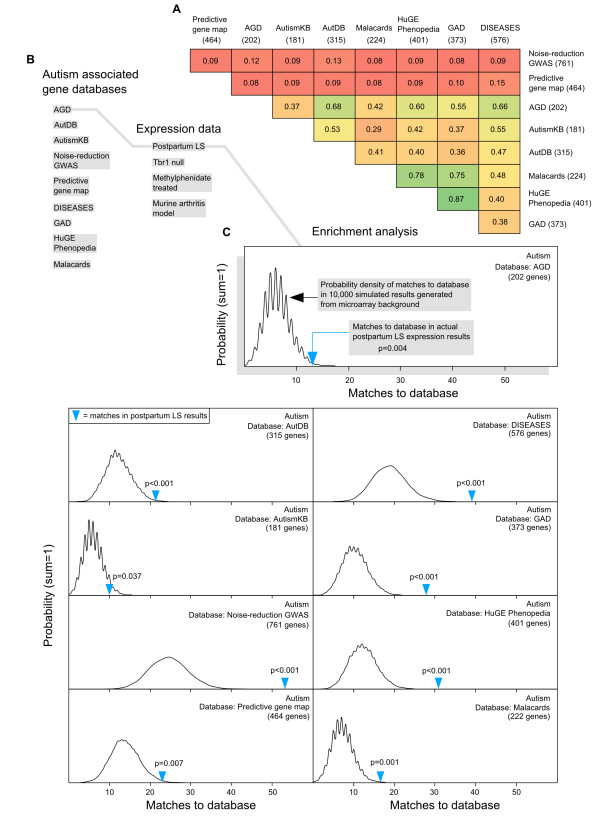
**Autism database comparison, modular workflow of MSET analysis, and autism enrichment evaluation of postpartum LS expression results. A)** Pairwise comparison of redundancy in gene lists extracted from autism databases, calculated as the proportion of the smaller list that is identical to the larger list. Dark green corresponds to the highest possible redundancy (1), and red represents the least redundancy (0). **B)** Schematic view of modules that can be combined in MSET analysis. Enrichment of any gene database in the left hand column can be assessed within any set of expression data from the right hand column. The grey path connecting modules highlights the specific combination whose output is shown in the enlarged window in panel C. **C)** Output of MSET analysis for nine autism databases within postpartum LS expression results. The first window displays enrichment of Autism Genetic Database (AGD) genes within postpartum LS expression data, and is enlarged to include explanatory labels that highlight the important features of the graphical output. Y-axis represents the probability of X matches to database found in a randomly generated set of simulated results from the microarray background. The blue arrow shows how many matches were found in the actual significant postpartum LS expression changes and where that number falls on the probability density distribution. The enrichment p-value is derived from the number of simulated results that contained at least as many matches to database as the actual results. The following windows show MSET enrichment analyses for the other eight autism databases within postpartum LS expression data.

Nine independent online databases were selected as sources for autism-associated genes (Additional file 1: Table S1). Of these, four are general disease association databases, three are autism-specific aggregations, and two are recent publications that generated candidate autism gene lists. The autism gene lists extracted from the selected databases exhibited varying sizes and degrees of similarity as assessed by pairwise comparisons, but are generally non-redundant. Figure 1 illustrates the relatedness of the autism databases and shows how they fit into the modular workflow of the enrichment analysis.

**Figure 2:**
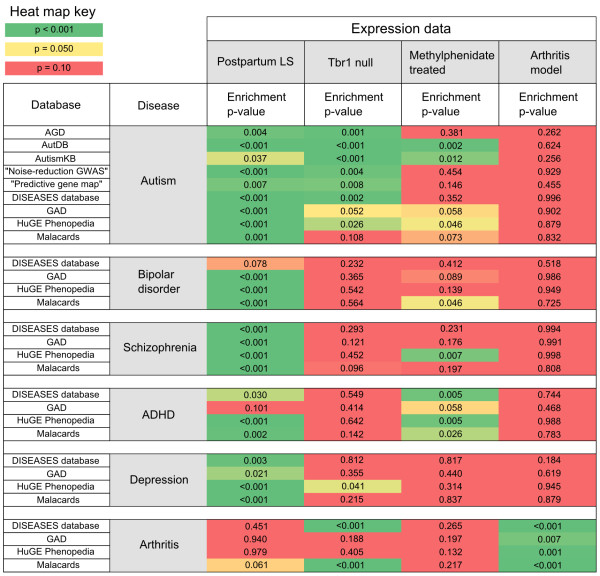
**MSET enrichment summary heat map for numerous disease-associated gene lists within multiple sets of expression data.** Gene lists representing each disease/disorder were extracted from databases shown in the far left column. Significance of enrichment p-values are mapped to a color spectrum as shown in the key (upper left). The four columns farthest to the right correspond to each set of expression data evaluated in this study.

A set of 809 genes displayed significant changes in expression in the postpartum LS relative to virgin (FDR-adjusted p < 0.25) as measured by microarray analysis. Within these results, enrichment for all nine autism gene lists was detected (Figure 1, p < 0.05). Collectively, 160 autism-associated genes were identified in the significant maternal LS expression results (Additional file 2: Table S2). To highlight the genes in this group that have the most evidence for an association to autism, 36 differentially expressed genes in the postpartum LS which appeared in three or more autism databases are shown in Table 1. A functional profile of all 160 autism-linked genes in the postpartum LS results generated by NIH’s DAVID functional annotation clustering tool revealed striking themes of differentiation/development, synaptic plasticity, and neuronal signaling (Table 2). Using gene lists extracted from the general disease association databases, enrichment was additionally discovered in the maternal LS for genes related to bipolar disorder, schizophrenia, ADHD, and depression. This is shown in Figure 2, which is a comprehensive heat map that summarizes enrichment results for all data sets analyzed in this study.

**Table 1:** Genes with strong links to autism found in significant postpartum LS expression changes

**Gene symbol**	**Gene name**	**Autism databases**
Foxp2	Forkhead box P2	7
Sez6l2	Seizure related 6 homolog like 2	7
Lamb1	Laminin B1	7
Slc1a1	Solute carrier family 1 (neuronal/epithelial high affinity glutamate transporter, system Xag), member 1	7
Adora2a	Adenosine A2a receptor	6
Gabra4	Gamma-aminobutyric acid (GABA) A receptor, subunit alpha 4	5
Hras1	Harvey rat sarcoma virus oncogene 1	5
Arnt2	Aryl hydrocarbon receptor nuclear translocator 2	5
Lrfn5	Leucine rich repeat and fibronectin type III domain containing 5	4
Scn1a	Sodium channel, voltage-gated, type I, alpha	4
Drd2	Dopamine receptor D2	4
Nostrin	Nitric oxide synthase trafficker	4
Npy	Neuropeptide Y	4
Snrpn	Small nuclear ribonucleoprotein N	4
Fabp5	Fatty acid binding protein 5, epidermal	4
Pcdh10	Protocadherin 10	4
Kcnd2	Potassium voltage-gated channel, Shal-related family, member 2	3
Upp2	Uridine phosphorylase 2	3
Rbfox1	RNA binding protein, fox-1 homolog (C. elegans) 1	3
Adra2a	Adrenergic receptor, alpha 2a	3
Cadps2	Ca2 + −dependent activator protein for secretion 2	3
Camk2b	Calcium/calmodulin-dependent protein kinase II, beta	3
Csmd3	CUB and Sushi multiple domains 3	3
Drd1a	Dopamine receptor D1A	3
Fabp7	Fatty acid binding protein 7, brain	3
Foxo1	Forkhead box O1	3
Hcrtr1	Hypocretin (orexin) receptor 1	3
Htr5a	5-hydroxytryptamine (serotonin) receptor 5A	3
Mchr1	Melanin-concentrating hormone receptor 1	3
Nr2e1	Nuclear receptor subfamily 2, group E, member 1	3
Oprk1	Opioid receptor, kappa 1	3
Pde4b	Phosphodiesterase 4B, cAMP specific	3
Ppp1r1b	Protein phosphatase 1, regulatory (inhibitor) subunit 1B	3
Rarb	Retinoic acid receptor, beta	3
Robo2	Roundabout homolog 2 (Drosophila)	3
Tac1	Tachykinin 1	3

All genes in Table 1 have expression changes with FDR-adjusted p-values less than 0.25. The farthest right hand column shows the number of autism databases in which the gene was featured, and represents a putative strength of evidence for a positive association to autism. Only genes that appeared in three or more lists are shown here. A full list of autism-associated genes from all databases found in postpartum LS expression changes is provided in Additional file 2: Table S2.

**Table 2:** NIH DAVID functional annotation clustering of autism-associated genes found to be significantly altered in postpartum LS

**Cluster**	**Enrichment score**	**Genes**				
Synapse/Cell Junction	7.69	Cadps	Ctnnb1	Gabrd	Pclo	Syt6
		Rasgrp2	Cplx2	Grik1	Scn1a	
		Snapin	Cpeb1	Grm3	Sv2c	
		Adora2a	Dlg1	Lrfn1	Synpr	
		Camk2a	Gabra4	Phactr1	Syt5	
Regulation of synaptic plasticity/transmission	4	Hras1	Drd1a			
		Adora2a	Grik1			
		Camk2a	Grm3			
		Cpeb1				
		Drd2				
Neuron projection morphogenesis	3.75	Dscam	Adora2a	Gas7		
		Klf7	Baiap2	Nr2e1		
		Sh2b1	Ctnnb1	Phgdh		
		Slitrk1	Cxcr4	Actg1		
		Alcam	Drd2	Robo2		
Cyclic nucleotide mediated signaling	3.35	Htr5a	Pclo			
		Adora2a	Tshr			
		Drd2				
		Drd1a				
		Gna11				
Cell morphogenesis/differentiation	2.73	Dscam	Cxcr4			
		Klf7	Drd2			
		Slitrk1	Nr2e1			
		Alcam	Robo2			
		Ctnnb1				
Neurogenesis/nervous system development	2.48	Ascl1	Smo			
		Ctnnb1	Robo2			
		Drd2	Socs2			
		Mapt				
		Nr2e1				
Regulation of glutamine/amine/GABA signaling	2.44	Adora2a				
		Drd2				
		Drd1a				
		Grik1				
		Nnat				
Cell motility/migration	2.4	Flt1	Drd1a			
		Ascl1	Nr2e1			
		Cxcr4	Pex5l			
		Cx3cl1	Smo			
		Drd2				

Enrichment scores were calculated for all 160 autism-associated genes found in significant postpartum LS results (FDR-adjusted p < 0.25, Additional file 2: Table 2) by NIH DAVID’s functional annotation clustering with “high” stringency setting selected.

**Figure 3:**
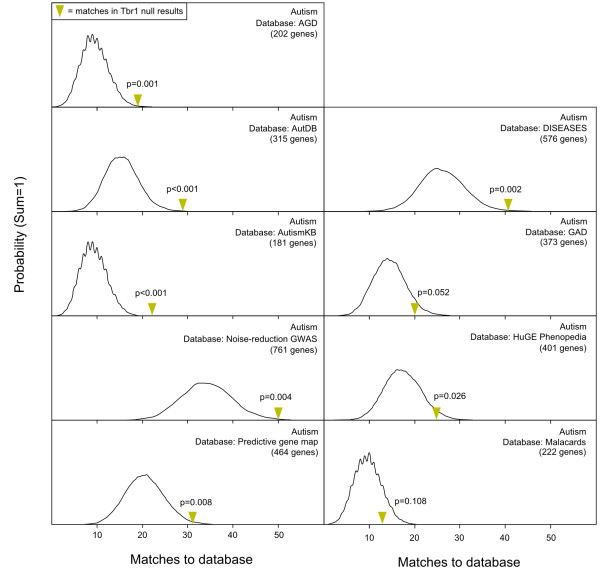
**MSET evaluation of enrichment for autism-associated genes within expression data from *Tbr1 *null mouse neocortex.** Y-axis represents the probability of X matches to database found in a randomly generated set of simulated results from the microarray background. The yellow arrow shows how many matches were found in the actual significant *Tbr1* null expression changes and where that number falls on the probability density distribution. The enrichment p-value is derived from the number of simulated results that contained at least as many matches to database as the actual results.

As a demonstration of MSET’s applicability, enrichment of autism gene lists was evaluated in a set of microarray results from the developing neocortex of T-box brain gene 1 (*Tbr1*) null mice, a putative model for autism genetics. In the 809 most significant results, these expression data exhibited compelling enrichment in seven out of the nine autism gene lists (P < 0.05), with subtler enrichment in two gene lists that bordered on or failed to reach significance (Figure 3). In contrast to the maternal LS results, the *Tbr1* data showed little to no enrichment in genes associated with other mental health disorders (Figure 2).

Furthermore, enrichment analysis for the full complement of mental health disorders was conducted within a set of 809 significantly altered genes in microarray results from mice treated with methylphenidate, a common treatment for ADHD. These expression data exhibited some enrichment for autism gene lists (two out of nine, p < 0.05), but notably showed high enrichment specifically for ADHD gene lists (Figure 2).

**Figure 4:**
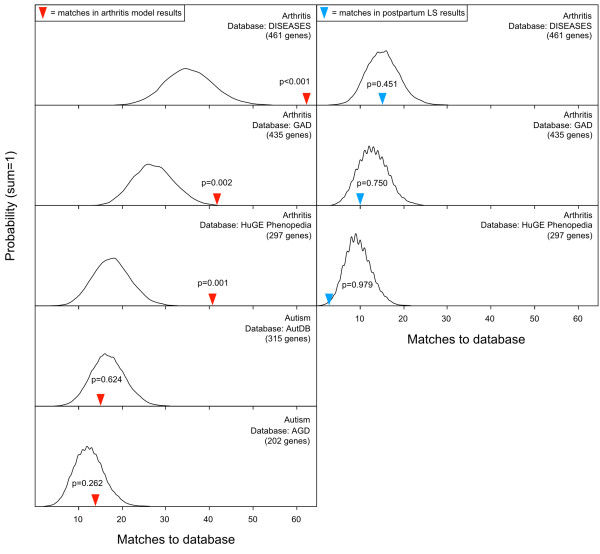
**MSET evaluation of enrichment for autism and arthritis-associated genes within expression data from a murine arthritis model and postpartum LS.** The left hand column of windows features the graphical output of MSET enrichment analyses for representative arthritis-associated gene lists (top three windows) and selected autism-associated gene lists (bottom two windows) within expression data from a mouse model of induced arthritis. The right hand column of windows shows the graphical assessment of enrichment for representative arthritis-associated genes within postpartum LS expression results. Y-axis represents the probability of X matches to database found in a randomly generated set of simulated results from the microarray background. The red arrow shows how many matches were found in the actual significant arthritis model expression changes and where that number falls on the probability density distribution, while the blue arrow represents matches in the actual significant postpartum LS expression results. The enrichment p-values are derived from the number of simulated results that contained at least as many matches to database as the actual results.

As a test for specificity of MSET analysis, enrichment for autism and arthritis-associated gene lists was compared within two sets of expression data; the 809 significant maternal LS microarray results and 809 microarray results from a serum induced murine arthritis model. Enrichment of arthritis-associated genes was detected in the arthritis model expression data, but not in the maternal LS results (p < 0.05). Additionally, enrichment of autism gene lists was not detected within the arthritis model results (Figure 4). The arthritis model results failed to show enrichment for any of the mental health disorders (Figure 2).

## Discussion

In this study, we have introduced the Modular Single-set Enrichment Test (MSET), a newly developed tool designed for assessing enrichment of disease/disorder-associated gene sets within microarray results. In addition to demonstrating the capabilities and limitations of this novel software, we used it to discover a strong link between the maternal brain and autism, as well as several other mental health disorders. We subsequently identified a network of candidate genes that may influence sociability in mothers and revealed the functional character of this network to be primarily related to developmental and neuronal signaling processes.

### Enrichment in postpartum LS for autism, and other mental health disorders

The compelling enrichment of autism-associated genes found in expression changes of the postpartum LS (Figure 1) is, to our knowledge, the first demonstration of a genetic link between the maternal brain and pathways involved in autism. The discovery of enrichment in postpartum LS for several mood/social disorders (Figure 2) suggests that the phenotypic consequence of LS gene changes in the transition to motherhood possesses a significant behavioral and emotional component. Because the mother-infant relationship is the first and foremost social bond formed in mammals, it has been suggested that the genetic and neural networks underlying sociability in this ancestral event might serve as an evolutionary template from which sociability in other contexts is derived [35]. Our data indirectly support this concept on a large-scale genetic level. While autism rates are higher in males [36], it could be the conserved use of the same core genes for sociability that provides the connection between autism and the maternal brain. Furthermore, the severity of autism symptoms is often described as spectral, rather than binary. It is therefore plausible that subtle dysregulation of genes which are naturally modulated in the control of sociability, such as in the transition to motherhood, would more likely contribute to this observed phenomenon than would rarer gain-of-function or loss-of-function mutations.

Table 1 presents 36 of the 160 autism-associated genes that MSET identified in the postpartum LS, as well as the number of autism databases in which they are featured. This is presumably a reflection of the strength of their association with autism based on past studies, with consensus genes having the most widely recognized evidence. However, it is not a perfect indicator because it only counts positive association discoveries, and does not consider the existence of any potential contradictory evidence or disagreement. For example, *Foxp2*, a forkhead/winged helix (FOX) transcription factor, is found in seven of the nine autism databases used (Table 1). It is located in a region of chromosome 7q that has been linked to autism in the past [37], and mutations in *Foxp2* cause speech and language acquisition pathologies in humans [38]. However, more recent evidence suggests that the language deficits are more directly related to a developmental impairment of motor brain regions, rather than to social behavior, and several recent reports conclude that *Foxp2* does not contribute to autism susceptibility [39-41]. Even if *Foxp2* were omitted from significant postpartum LS expression results, the observed enrichment would be highly significant. This illustrates the important point that, although assessing the degree of enrichment using MSET is robust and largely resistant to single gene false positives in upstream databases, caution must be exercised when interpreting the biological importance of individual genes identified by MSET in the testing procedure. Another advantage of MSET is that the user can manually annotate any file, remove genes that are considered to be inappropriate, or even create novel gene lists for testing.

Table 1 includes several autism-linked genes that were identified in our original microarray analysis as particularly interesting based on their biological function and relevance to emotional state and behavior. These include the GABA_A_ receptor subunits α4 and δ, four potassium channel subunits (*Kcnd2*, *Kcnd3*, *Kcnh7*, and *Kcnj4*), dopamine receptors *Drd1a* and *Drd2*, the kappa opioid receptor *Oprk1*, fatty acid binding protein 7 (*Fabp7*), and suppressor of cytokine signaling 2 (*Socs2*). The biology of these genes is discussed in greater detail in our original report [11].

NIH DAVID’s functional annotation clustering tool was used to generate a functional profile of the 160 autism-associated genes found to be differentially expressed in the postpartum LS (Table 2). The most highly enriched pathways were primarily developmental, involving processes such as synaptic plasticity, neuronal morphogenesis/differentiation, and cell motility. Several clusters related to synaptic transmission also showed high levels of enrichment. Because these biological processes have now been implicated in both autism and the maternal LS, it is likely that aspects of sociability modulated in both phenomena are influenced by structural changes in the brain, including axonal/dendritic growth, and even neurogenesis. This possibility is supported by a body of literature which has revealed that diverse regions of the adult brain contain multipotent stem cells capable of generating new neurons [42-49], and it has been shown that maternal behavior is associated with the stimulation of neurogenesis in the subventricular zone [50].

In addition to autism, MSET analysis revealed that significant postpartum LS expression results exhibit enrichment for bipolar disorder (BPD), schizophrenia, ADHD, and depression-associated genes (Figure 2). These gene lists were extracted from the four general disease association databases that were also used in the autism enrichment analysis (Additional file 1: Table S1). Links were particularly strong for both BPD and schizophrenia. BPD and depression links are of interest because rates of depression increase in the postpartum state, with postpartum depression affecting 1-10% of mothers [51]. Thus, some of the normal changes that occur in the maternal brain likely lead to a vulnerability of key depression type pathways. Positive associations have been consistently found for an elevated risk of BPD in women after childbirth [52], which is considered to be part of a suite of diagnosable “postpartum psychoses”. Also among this class of diseases is schizophrenia, which, in addition to its well-known cognitive dysfunction, is also characterized by emotional deficits [53]. Recent studies highlight that a subset of genes contribute to multiple mental health disorders [54-57], so it is not completely surprising that a behavioral transformation as fundamental as the transition to motherhood might have links to multiple disorders. To ensure that this multitude of positive enrichment was not due to an artifact in MSET analysis, we tested the postpartum LS expression results for enrichment of arthritis-associated genes (Figure 4), which proved to be absent. The MSET tool has been used successfully in our laboratory to detect enrichment of mental health-related gene sets in other areas within the maternal brain, such as the medial preoptic area (unpublished observations). While there were similarities in enrichment across regions, there were also differences in enrichment patterns and in the individual genes which accounted for enrichment. This indicates that there may be common, global expression changes in the maternal brain, but also that each region has its own genetic “signature”. Future work will characterize the genetic profile of the maternal brain more comprehensively.

### Enrichment analysis in expression data from a murine model of induced arthritis

To validate and demonstrate the applicability of MSET, we performed a series of analyses on expression data taken from several independently conducted microarray experiments. These data are publicly available through NCBI’s Gene Expression Omnibus (http://www.ncbi.nlm.nih.gov/geo/) or through institutional hosting.

To test for expected specificity of MSET analysis, we assessed enrichment for the full range of disease-associated gene sets within microarray expression data from a murine arthritis model study in which an arthritic state was induced via the transfer of serum from a knockout mouse into a wild type animal [58]. Complementary findings to the postpartum LS results were observed, in which the arthritis model data showed enrichment specifically for arthritis-associated gene sets, but not for autism (Figure 4) or any other mental health disorders (Figure 2). Collectively, these results demonstrate that the enrichment analysis performed in the present study is reliable and specific. Specificity may not be expected in every application, as different models and experimental treatments used in microarray studies can affect broad or unanticipated gene pathways.

### Enrichment of autism-associated genes in Tbr1 null transgenic mice

To showcase the broader applicability of MSET, we performed enrichment analysis for the full range of disease-associated gene lists in a set of expression data collected from murine T-box brain gene 1 (*Tbr1*) null developing neocortex [59]. The *Tbr1* null animal was chosen because *Tbr1* is a developmentally related transcription factor that binds, among other targets, the promoter of a gene called autism susceptibility candidate 2 (*Auts2*), named for its implication in autism susceptibility in the frontal cortex [59,60]. The *Tbr1* null neocortex was observed to be enriched specifically for autism-associated gene sets (Figure 3), and not for any other mental health disorders included in our analysis (Figure 2). These findings suggest that, although inviable shortly after birth, the *Tbr1* knockout animal may provide a valuable model for the study of autism-related biology. The *Tbr1* null expression data also showed enrichment in two out of the four arthritis-associated gene sets. While this is not particularly strong enrichment, the observed variability could be due to broader physiological changes across numerous systems (possibly including the immune response) that must undoubtedly be affected by the fatal null mutation.

### MSET enrichment analysis in expression data from methylphenidate treated mice

In addition to using MSET to analyze enrichment in expression results from animals that have undergone a natural change (mothers) and transgenic animals (*Tbr1* null), we also tested its capabilities in a set of expression data from mice that were subjected to a pharmacological treatment. In the study, mice were treated with chronic (90 days) exposure to methylphenidate, commonly used to treat ADHD, and microarray analysis was performed on microdissected substantia nigra pars compacta (SNpc) [61]. In our enrichment analysis of these data, we observed a subtle degree of enrichment for autism-related gene sets (in three out of nine lists), but found that a consensus of enrichment was only detected for ADHD-related gene lists, and not for any other mental health disorder or arthritis (Figure 2). This shows that MSET can be effectively utilized with sensitivity in microarray data collected from a variety of different experimental protocols and treatments, providing a promising new strategy for exploring the genetics underlying mental health disorders from numerous, complementary angles.

### Considerations and limitations of MSET analysis

MSET allows for powerful research possibilities, but there are numerous considerations that must be made regarding its appropriate application and the input parameters used. MSET utilizes a fairly simple gene randomization testing procedure to determine if members of a disease-associated gene set are overrepresented within significant microarray results compared to what would be expected by chance. This is in contrast to programs like GSEA, in which the coincident distribution of gene set members is characterized within a ranked list of microarray results using a running-sum statistic and correlated to phenotype with individual sample expression values [9]. Accordingly, MSET calls for only one simple input file of summarized microarray gene results (in addition to disease-associated gene sets of interest), and does not require expression values, chip annotations, or phenotype/trait files. Some web applications exist for performing overrepresentation analysis (such as GOHyperGAll in Bioconductor, InnateDB, and GenMAPP-CS in the GO-Elite program), but they include problematic gene ID conversions, species limitations, a strict dependence on GO terms and existing ontologies, and inflexibility in generating custom gene sets. MSET represents an advancement in versatility and ease of use over the existing landscape of tools for testing enrichment of independently curated disease-associated gene sets.

While MSET is theoretically capable of testing for enrichment of genes linked to functional pathways, using other, more full-featured programs for this purpose is recommended. It has been proposed that gene independence is a safe assumption for enrichment analysis [62]. Others have countered that gene-gene interaction can inflate p-values and generate false positives in functional enrichment. Due to the relatively simple nature of the randomization algorithm, independence is assumed in MSET analysis for disease enrichment. This is a safe assumption because disease gene sets are heterogeneous groups curated by phenotypic associations, rather than functional relatedness. However, it would be more conservative to use other programs that account for potential gene-gene interaction in functional enrichment analyses. GSEA, for instance, preserves gene-gene interaction by permuting labels of whole samples, rather than at the individual gene level [9].

Because enrichment analysis is highly sensitive to the input gene lists used [1], this will be a focus for much of the discussion regarding the performance of MSET. It can be seen in Figure 1 that the nine autism databases used in our analysis vary in both their identity and size. They also differ in the methods used to produce candidate autism gene lists; therefore, one should be aware that some databases may be more robustly assembled than others, and confidence in MSET results relies critically on confidence in upstream database quality. MSET’s reliability is bolstered by its capacity to test enrichment for multiple gene lists associated with the same disease; this feature minimizes the effects of weak associations on enrichment testing significance.

There is generally a balance between specificity of enrichment and the accuracy of its detection, which is related to gene list length. This is particularly relevant when extracting gene lists from general disease association databases (such as the DISEASES database, GAD, HuGE Phenopedia, and Malacards), which compile positive associations broadly across many diseases. Smaller gene lists may be more specific to their associated disease, but the MSET suffers from a decrease in the accuracy of hypothesis testing as the average number of matches found in simulated results becomes small. This can be seen in the probability density curves for database matches generated with the AutismKB and Malacards autism gene lists in Figure 1. Their “spikey” appearance reflects the highly discrete nature of distributions with a very small range. Consequently, chance variation in the number of matches in the microarray results being analyzed, even by a single gene, represents a disproportionately large jump in p-value from peak to peak. The smoother distributions generated from larger databases provide a much greater resolution for hypothesis testing; however, larger gene lists may be less biologically specific to the associated disease, and extremely large gene lists can result in false positive enrichment results. While there is assuredly some “true” degree of genetic overlap underlying various diseases, there is probably an additional level of similarity across seemingly unrelated conditions introduced artificially through the methodology of association studies and their aggregation. For example, one might expect genes featured in centrally important signaling pathways to show positive associations with many diseases and experimental conditions in microarray studies, leading to false positive results for enrichment of extremely large gene lists. Specificity can be further complicated by the detailed nature of disease association labels in comprehensive databases. For instance, the DISEASES database has separate gene lists for arthritis, psoriatic arthritis, osteoarthritis, rheumatoid arthritis, septic arthritis, and more. The primary capacity of MSET to overcome these factors is rooted in its ability to be repeated modularly to generate a meta-analysis. This allows for isolated enrichment findings to be interpreted within the context of larger patterns. Also, because a deeper and more refined body of resources exists for autism genetics than for the other disorders featured in this study, we have relatively greater confidence in the downstream autism enrichment results. As ongoing research adds to our genetic understanding of various diseases, the MSET tool is in an ideal position to allow researchers to swiftly adapt and make use of updated knowledge bases in the future.

The tradeoff between specificity and accuracy also applies to the significant microarray results in which enrichment testing is performed. For postpartum LS expression data, we used an FDR-adjusted p-value of 0.25 as a significance threshold, which produced 809 genes from the microarray background. Researchers using other model organisms or biological systems may want to use different criteria for statistical significance. Other microarray studies may not yield a high number of significant gene changes by FDR-adjusted p-values. In these situations, a less stringent significance threshold may be applied to make use of a larger number of results, but the greater inclusivity and incidence of false discovery may render them somewhat less biologically meaningful. The subsequent enrichment analysis must therefore be taken with an accordingly critical interpretation.

MSET is designed to allow the user to conduct the most appropriate examination possible for enrichment of one or more disorders in a particular set of expression data. Due to the necessarily customizable nature of the input parameters that make for a quality assessment in one set of microarray results, it is difficult to objectively compare enrichment across numerous expression results. In the current study, we have done so by standardizing both the number of significant expression results selected from the background and the databases used. It cannot be assumed that the 809 most significant genes from one study are as meaningful or specific as those from another study, but the comprehensive and identically repeated analysis performed here is a valuable preliminary comparison. Collectively, the analyses undertaken in the current study provide a promising indication that the MSET method can be a valuable and informative approach to large scale genetic questions.

## Conclusions

In this article, we present the novel enrichment tool MSET as a lightweight alternative for performing enrichment analysis with custom-built, disease-associated gene lists. The value of MSET is twofold; firstly in its ease of use for researchers of any background due to its methodological simplicity and transparency, and secondly in the statistical hypothesis testing power offered by the ability to test enrichment within expression data modularly with numerous, independent gene-association databases. Using the MSET approach, we discovered that the maternal LS is highly enriched for autism-associated genes, as well as for genes linked to related mental health disorders, thus supporting a role for the maternal brain as a valuable translational model to the genetics of social and emotional disorders. By matching genes within significant expression results to autism genetic databases, we constructed a network of candidate genes that may regulate sociability in new mothers as well as in contexts of psychiatric illness. We further revealed that this network is primarily related to developmental processes, indicating that structural changes in LS likely underlie the modulation of social interaction and emotional reactivity in the postpartum state.

## Availability and requirements

The Modular Single-set Enrichment Test (MSET) is available for download as Additional file 3, and is hosted online at https://sourceforge.net/projects/mset2013. It is written in R and requires the R command console to run, which can be downloaded from http://www.r-project.org/. It is licensed under the open source Apache 2.0 license. Refer to the MSET manual contained within the MSET folder for instructions on installation and use.

## Abbreviations

MSET: Multiple single-set enrichment test; LS: Lateral septum; ADHD: Attention deficit hyperactivity disorder; BPD: Bipolar disorder; GSEA: Gene set enrichment analysis; DAVID: Database for annotation, visualization and integrated discovery; GO: Gene ontology; KEGG: Kyoto encyclopedia of genes and genomes; OMIM: Online mendelian inheritance in man; GAD: Genetic association database; AGD: Autism genetic database; GEO: Gene expression omnibus.

## Competing interests

The authors declare that they have no competing interests.

## Authors’ contributions

BE, MS, TD, and SG conceived and developed the MSET script. BE carried out MSET validation on independent microarray data sets and performed MSET analysis on postpartum LS expression data. BE and SG drafted the manuscript. All authors read and approved the final manuscript.

## Supplementary Material

Additional file 1: Table S1 Source information for the disease and autism association genetic databases used in this study. Click here for file

Additional file 2: Table S2 Autism-associated genes found in significant (FDR-adjusted p < 0.25) postpartum LS expression results. Click here for file

Additional file 3 MSET Folder containing MSET script and associated files. Click here for file
