# Supplementary material for: Development of a versatile enrichment analysis tool reveals associations between the maternal brain and mental health disorders, including autism
Source: BMC Neurosci. 2013 Nov 19;14:147. doi: 10.1186/1471-2202-14-147 (PMC3840590; doi:10.1186/1471-2202-14-147)
Supplement: Additional file 1: Table S1 — Source information for the disease and autism association genetic databases used in this study. [file 1471-2202-14-147-S1.docx]

| **Supplementary Table 1. Source information for the disease and autism association genetic databases used in this study.** | | | | |
| --- | --- | --- | --- | --- |
| **Source** | **Description** | **Number of genes** | **Link** | **Comments** |
| DISEASES – The Novo Nordisk Foundation Center for Protein Research at the University of Copenhagen | Disease-gene associations mined from literature | 576 | http://diseases.jensenlab.org/Search | Selected genes associated with “Autistic disorder”, [DOID:12849] |
| Genetic Association Database (GAD) – Center for Information Technology, National Institutes of Health | Human genetic association studies of complex diseases and disorders | 373 | http://geneticassociationdb.nih.gov/cgi-bin/index.cgi | Selected genes associated with “autism” phenotype |
| HuGE Navigator Phenopedia – Centers for Disease Control and Prevention | An integrated, searchable knowledge base of genetic associations and human genome epidemiology | 401 | http://www.hugenavigator.net/HuGENavigator/home.do | Selected genes associated with “Autistic disorder” |
| Malacards – Crown Human Genomics Center and the Bioinformatics Unit of the Weizmann Institute of Science | An integrated database of human maladies and their annotations, modeled on the architecture and richness of the popular GeneCards database of human genes | 224 | http://www.malacards.org/ | Selected genes associated with “Autism spectrum disorder” |
| AutDB - MindSpec | An integrated catalogue of human genetic studies related to autism | 315 | http://autism.mindspec.org/autdb/Welcome.do | Selected human genes |
| AutismKB – Center for Bioinformatics, Peking University | An evidence based knowledgebase of autism genetics | 181 | http://autismkb.cbi.pku.edu.cn/ | Combined “Core” and “Syndromic” categories |
| Autism Genetic Database (AGD) | A comprehensive database for autism susceptibility gene-CNVs integrated with known noncoding RNAs and fragile sites | 202 | http://wren.bcf.ku.edu/ | Selected all genes |
| “A Brain Region-Specific Predictive Gene Map for Autism Derived by Profiling a Reference Gene Set” | Autism candidate genes generated by filtering the human genome with expression/functional profiles of 84 significant autism susceptibility genes from AutDB | 464 | http://www.plosone.org/article/authors/info%3Adoi%2F10.1371%2Fjournal.pone.0028431 | Selected Table S9. Set of 460 |
| “A noise-reduction GWAS analysis implicates altered regulation of neurite outgrowth and guidance in autism” | Autism candidate genes generated by human GWAS analysis with statistical noise reduction to decrease false positives | 761 | http://www.molecularautism.com/content/2/1/1 | Selected Table S4. |
