# Supplementary material for: Development of a versatile enrichment analysis tool reveals associations between the maternal brain and mental health disorders, including autism
Source: BMC Neurosci. 2013 Nov 19;14:147. doi: 10.1186/1471-2202-14-147 (PMC3840590; doi:10.1186/1471-2202-14-147)
Supplement: Additional file 2: Table S2 — Autism-associated genes found in significant (FDR-adjusted p < 0.25) postpartum LS expression results. [file 1471-2202-14-147-S2.docx]

| **Supplementary Table 2. Autism associated genes identified within significant postpartum LS expression changes.** | | | | | | | |
| --- | --- | --- | --- | --- | --- | --- | --- |
| Accn4 | Camk2a | Ddah1 | Foxp2 | Kcnd3 | Nell1 | Ppp1r1b | Snrpn |
| Actb | Camk2b | Ddx18 | Fstl5 | Kcnh7 | Nnat | Ptprn2 | Socs2 |
| Actg1 | Camta1 | Dlg1 | Gabra4 | Kcnj4 | Nostrin | Rarb | Sorcs2 |
| Adcyap1 | Car10 | Dner | Gabrd | Kif21a | Npy | Rasgrp2 | Spata13 |
| Adora2a | Cars | Dnm3 | Gas7 | Klf7 | Nr2e1 | Rbfox1 | Sv2c |
| Adra2a | Cdc42ep4 | Drd1a | Gdf10 | Lamb1 | Oprk1 | Rfk | Synpr |
| Adra2c | Cdca7 | Drd2 | Gna11 | Ldlrad3 | Parl | Rfx4 | Syt5 |
| Alcam | Cdh19 | Drp2 | Gpr176 | Lingo2 | Pbx3 | Rgs9 | Syt6 |
| Aplp1 | Cdk14 | Dscam | Gpr56 | Lphn2 | Pcdh10 | Rnd3 | Tac1 |
| Arid1b | Cntnap3 | Elovl6 | Grik1 | Lrfn1 | Pclo | Robo2 | Tbl1xr1 |
| Arnt2 | Cpa1 | Etl4 | Grm3 | Lrfn5 | Pde4b | Rxrg | Tet1 |
| Ascl1 | Cpeb1 | Fabp5 | H3f3b | Lrrn2 | Pde7b | Scn1a | Thsd7a |
| Atp2b1 | Cplx2 | Fabp7 | Hcrtr1 | Lzts2 | Peg10 | Sez6l2 | Tmem132b |
| Atp8a2 | Crispld2 | Fam43a | Hipk2 | Mapt | Pex5l | Sh2b1 | Tshr |
| Atpbd4 | Csmd3 | Fam49a | Hpcal4 | Maz | Phactr1 | Sh3rf2 | Tsnax |
| Baiap2 | Ctnnb1 | Farp1 | Hras1 | Mcc | Phgdh | Slc1a1 | Tubb2b |
| Bicd1 | Cx3cl1 | Flrt3 | Htr5a | Mchr1 | Plcb1 | Slc8a1 | Upp2 |
| Bmper | Cxcr4 | Flt1 | Iqgap2 | Meis2 | Plcd1 | Slitrk1 | Vgf |
| Cacng4 | Cxcr7 | Foxo1 | Itm2c | Ncam1 | Ppap2b | Smo | Ypel4 |
| Cadps | Dbpht2 | Foxp1 | Kcnd2 | Nedd4l | Ppapdc1a | Snapin | Zfhx3 |

All genes in Supplementary Table 2 have expression changes with FDR-adjusted p-values less than 0.25. In contrast with Table 1, which presents a subset of differentially regulated genes in the maternal LS that appear in three or more autism association lists, this table includes all 160 autism-linked genes found within the postpartum LS microarray results.
