# Supplementary material for: Development of a versatile enrichment analysis tool reveals associations between the maternal brain and mental health disorders, including autism
Source: BMC Neurosci. 2013 Nov 19;14:147. doi: 10.1186/1471-2202-14-147 (PMC3840590; doi:10.1186/1471-2202-14-147)
Supplement: Additional file 3 — MSET Folder containing MSET script and associated files. [file 1471-2202-14-147-S3.zip › MSET/MSET Manual.pdf]

Modular Single-set Enrichment Test (MSET) manual

Created at the University of Wisconsin-Madison (2013)
For questions, contact Brian Eisinger (beisin85@gmail.com)

If you wish to include MSET analysis/results in your publication, be sure to cite the original article that introduces the MSET approach:

Development of a versatile enrichment analysis tool reveals associations between the maternal brain and mental health disorders, including autism
Brian E Eisinger, Michael C Saul, Terri M Driessen and Stephen C Gammie
BMC Neuroscience 2013, **14**:147 doi:10.1186/1471-2202-14-147
Open access: http://www.biomedcentral.com/1471-2202/14/147/abstract

Table of contents

I. Introduction…….......2
II. The R language……….3
III. The MSET folder…….4
IV. Preparing files for use in MSET…………………………………………………………………………………………..5
V. Conducting MSET analysis………………………………………………………………………………………………..11

I. Introduction

The Modular Single-set Enrichment Test (MSET) is a simple, versatile randomization testing R script that allows researchers to assess enrichment for any gene list of interest within any set of expression results. In contrast to full-featured software that uses pre-set functional ontologies to search for and score enrichment across many biological pathways, the power of MSET stems from its ability to rigorously test the significance of enrichment for a single phenomenon of interest using independently curated gene association databases as "modules". The primary application of this approach is to analyze enrichment of disease and disorder associated gene sets, which are currently underrepresented in enrichment software. MSET serves as a tool to link previously disconnected resources in the biomedical and genetic communities with aims to (a) perform more thorough, high confidence enrichment tests within expression results for disease associated genes, and (b) to identify genes of interest in expression studies.

This instructional guide will illustrate in a step-by-step fashion how to create gene lists for use in MSET, how to conduct an MSET analysis, and how to read and interpret the results.

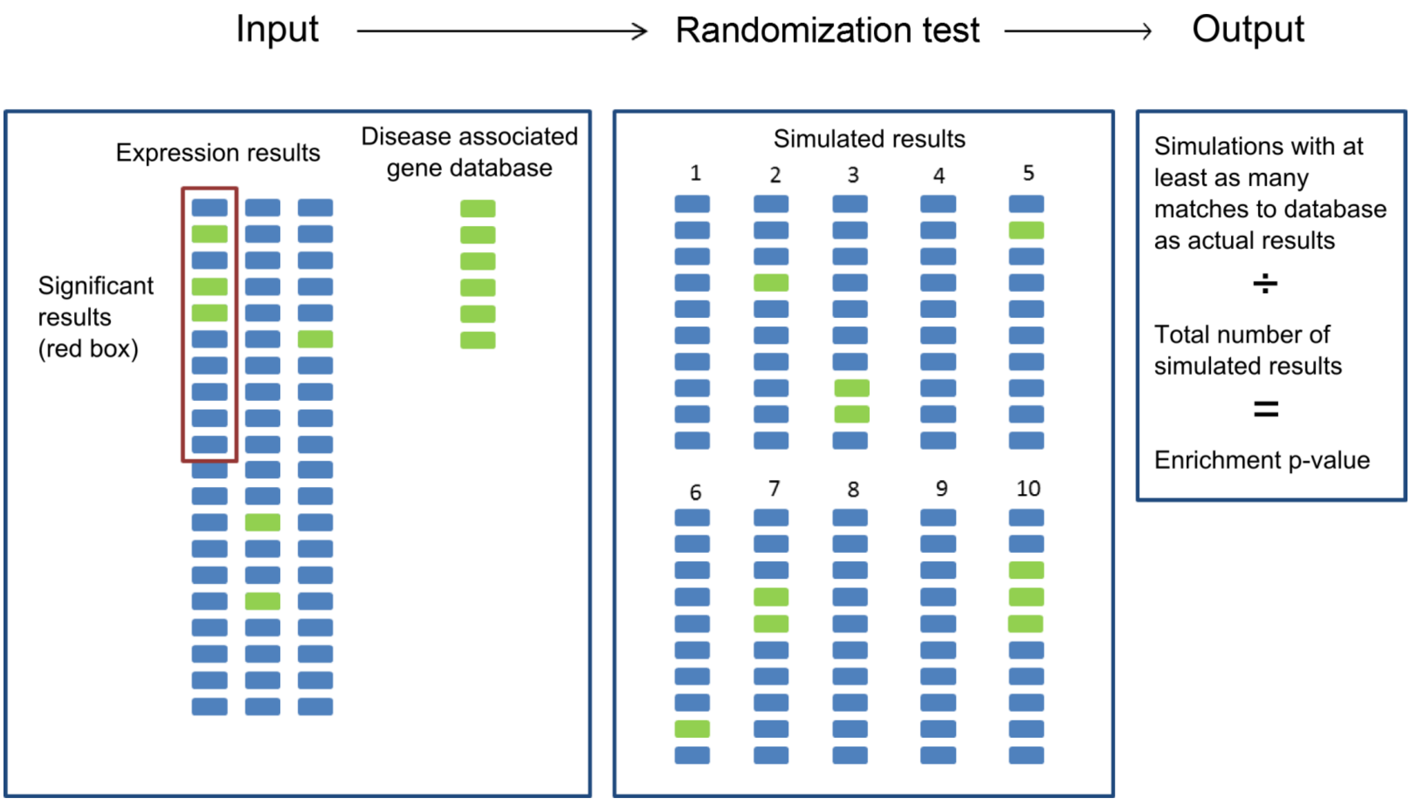

Simplified schematic of MSET's randomization testing method. MSET generates "simulated results" by sampling randomly and without replacement thousands of times from a microarray background, then calculates the probability of observing as many disease associated genes by chance as were seen in expression results from an actual experiment.

II. The R language

A. MSET is written in R, and is currently not hosted online. Consequently, the user must download the R client from http://www.r-project.org/ and run MSET through the command console.

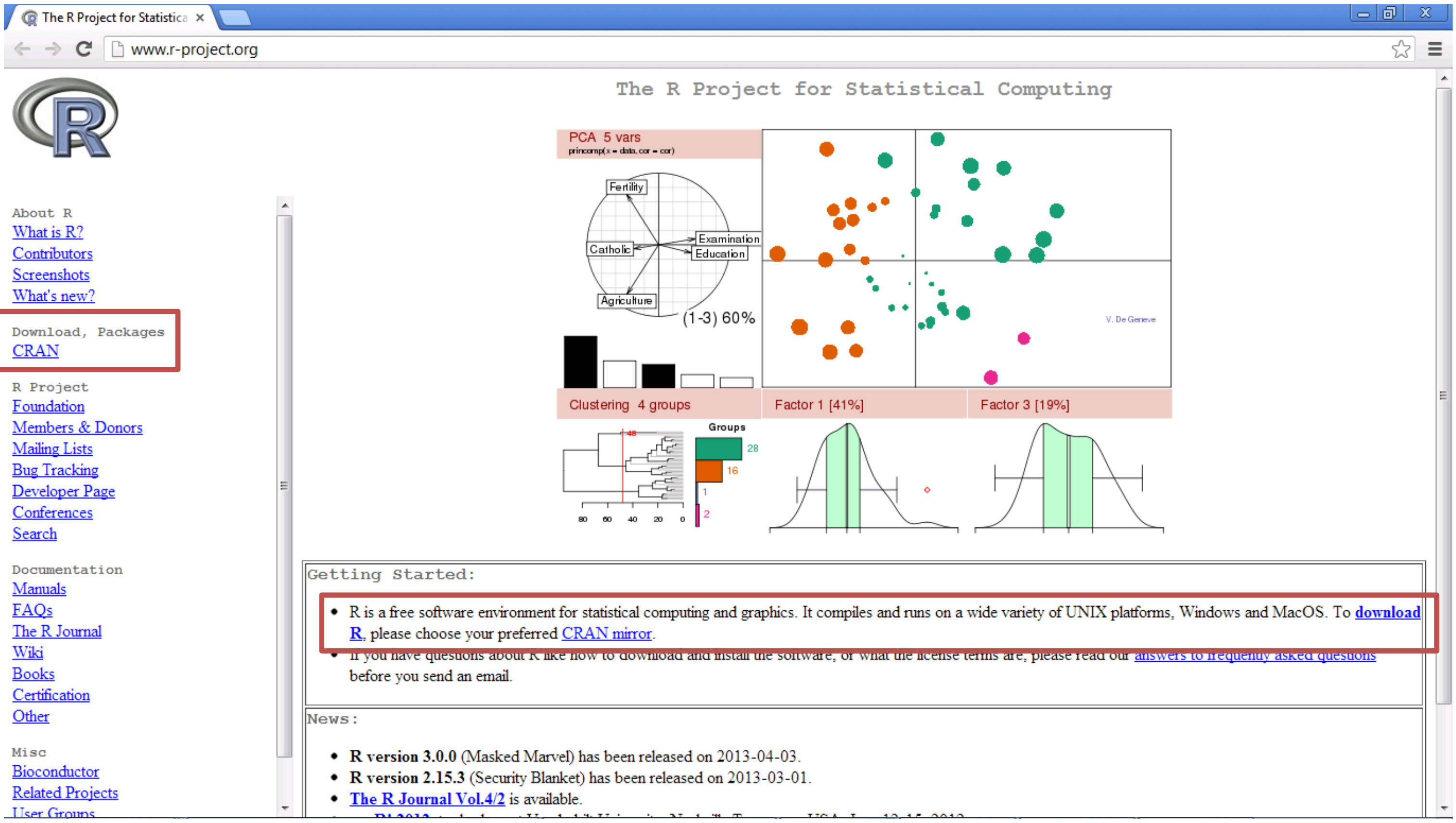

Links for downloading R can be found on the R website, in the areas highlighted by red boxes (current as of 2013).

III. MSET folder

The MSET folder must be downloaded from https://sourceforge.net/projects/mset2013/. The MSET folder includes the following files, as well as operating files that are generated by the MSET script and called upon later:

A. R scripts:
   a. `mset.R` – This is the MSET script.
   b. `hugo to mgi.R` – This script converts human gene lists (HUGO nomenclature) into mouse/rat (MGI) format.
   c. `mgi to hugo.R` – This script converts mouse gene lists (MGI nomenclature) into human (HUGO) format.

B. Annotation resources for nomenclature conversions
   a. `annotation key for HUGO and MGI.TSV` – This is the annotation key that the two converter tools ("hugo to mgi.R" and "mgi to hugo.R") use to find orthologous genes between species.

C. Example modules for learning and demonstration
   a. `postpartum LS results expression data MGI.txt` – This is a set of microarray expression results from a recently performed experiment comparing lateral septum (LS) in postpartum mice to virgin mice.
   b. `autism DISEASES MGI.txt` – This is a list of autism associated genes taken from the DISEASES database (http://diseases.jensenlab.org/Search).
   c. `autism HUGE phenopedia MGI.txt` – This is a list of autism associated genes taken from the HUGE Phenopedia (http://www.hugenavigator.net/HuGENavigator/startPagePhenoPedia.do).
   d. `autism malacards MGI.txt` – This is a list of autism associated genes taken from Malacards (http://www.malacards.org/).

III. Preparing files for use in MSET

MSET requires two input files:

1. One or more disease/disorder associated gene lists
2. One set of gene expression results
    a. This list includes the *full* set of expression results generated from a microarray experiment, including significant and non-significant changes, ordered by significance.

All disease associated gene lists and expression data sets used in MSET should be formatted as text files (.txt) containing gene identifiers, typically gene symbols, arranged in a single column with no column title. MSET operates by matching objects between input lists; consequently, the user is free to use any platform or species specific gene ID system. However, it is essential that the nomenclature is consistent for all input files.

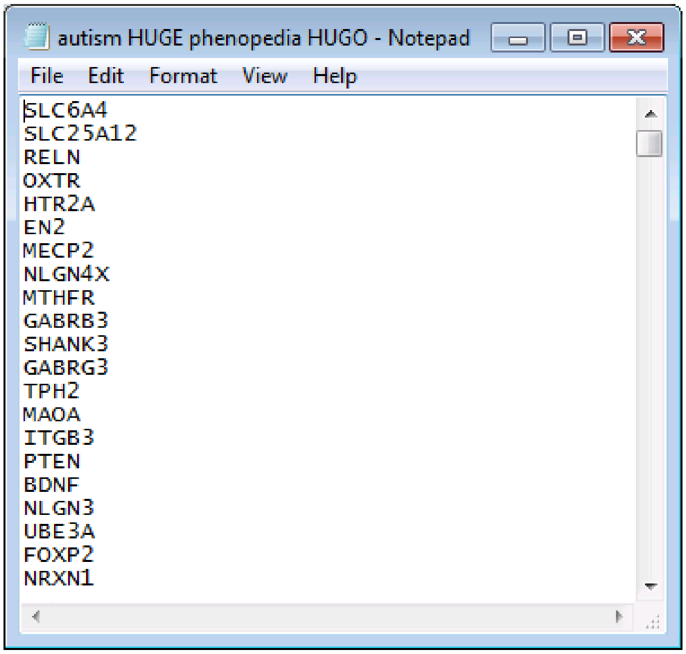

This is a list of autism linked genes from the HUGE Phenopedia. It is a single column of human (HUGO) format gene symbols.

Disease associated gene lists are most commonly available as human (HUGO) gene symbols. If the user's expression data are from non-human animals, the disease associated gene list will need to be converted into the appropriate species specific ID system to match. To easily perform conversions to mouse or rat (MGI) IDs, a tool is provided in the MSET folder. To convert gene symbols to other species, numerous online tools are available, including MADGene (http://cardioserve.nantes.inserm.fr/madtools/madgene/batch.php). To use the built-in HUGO/MGI conversion tool, first launch R from its installation location.

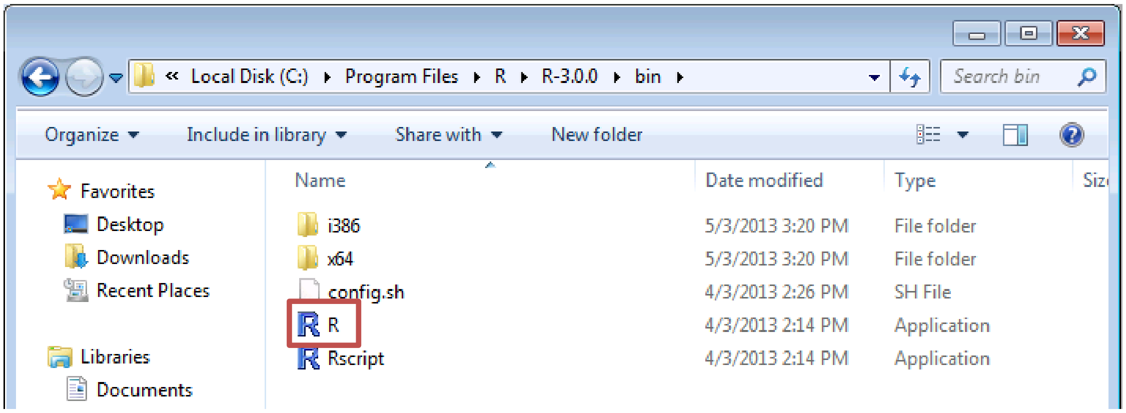

Launch R by executing the application from its installation location (red box).

Next, the MSET folder must be selected as R's working directory.

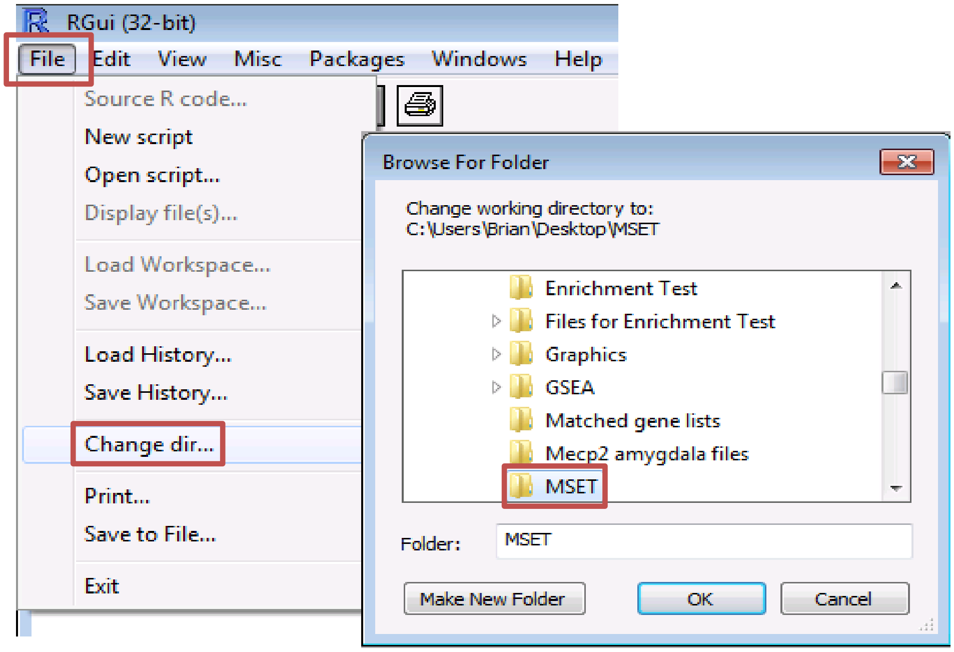

To designate the MSET folder as the working directory, click "File", "Change dir…", and select the MSET folder from its location on the hard drive. These steps are highlighted by red boxes.

After the MSET folder has been set as R's working directory, the gene conversion tool can be launched by typing `source("hugo to mgi.R")` in the command console and pressing ENTER.

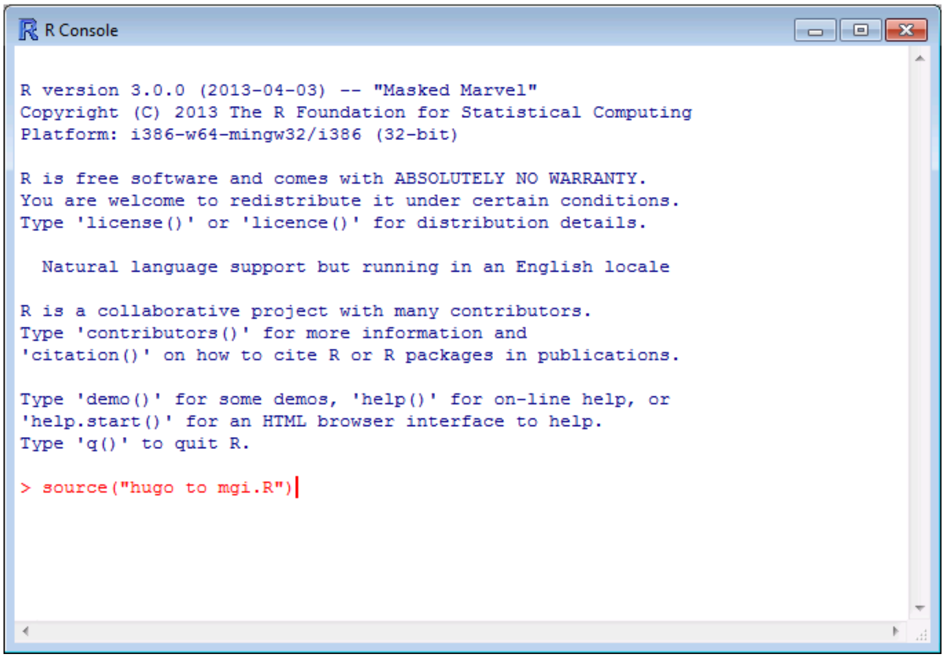

Launch gene ID conversion tool in the R console with the appropriate command line.

When prompted, select the text file of HUGO gene IDs to be converted to MGI.

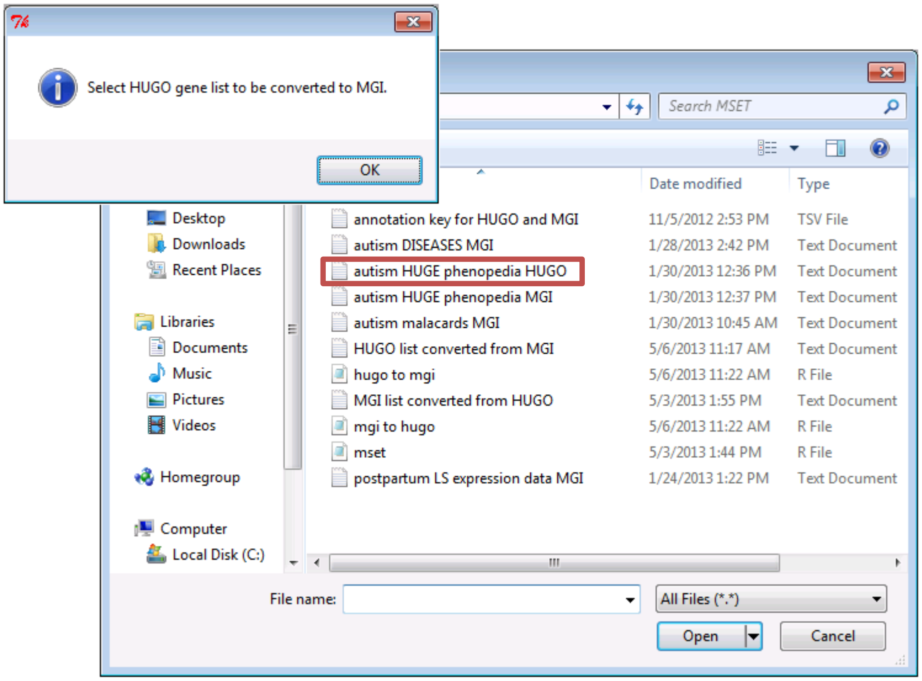

It is helpful to add "HUGO" or "MGI" in file names to see at a glance what format they are in. Select the HUGO gene list from the window prompt (red box).

The conversion tool then runs, and after a brief moment a text summary is displayed. It gives information about the proportion of the HUGO genes that were successfully converted into orthologous MGI gene IDs. This tool generally achieves > 90% conversion. Converting MGI to HUGO is also possible by using the command line `source("mgi to hugo.R")`. It is recommended that the user convert disease associated gene sets to match expression results, rather than vice versa, because expression results typically contain tens of thousands of genes. Even a 90% conversion rate from one species to another could result in a significant loss of data when applied to large gene lists. Converting smaller lists minimizes this possibility.

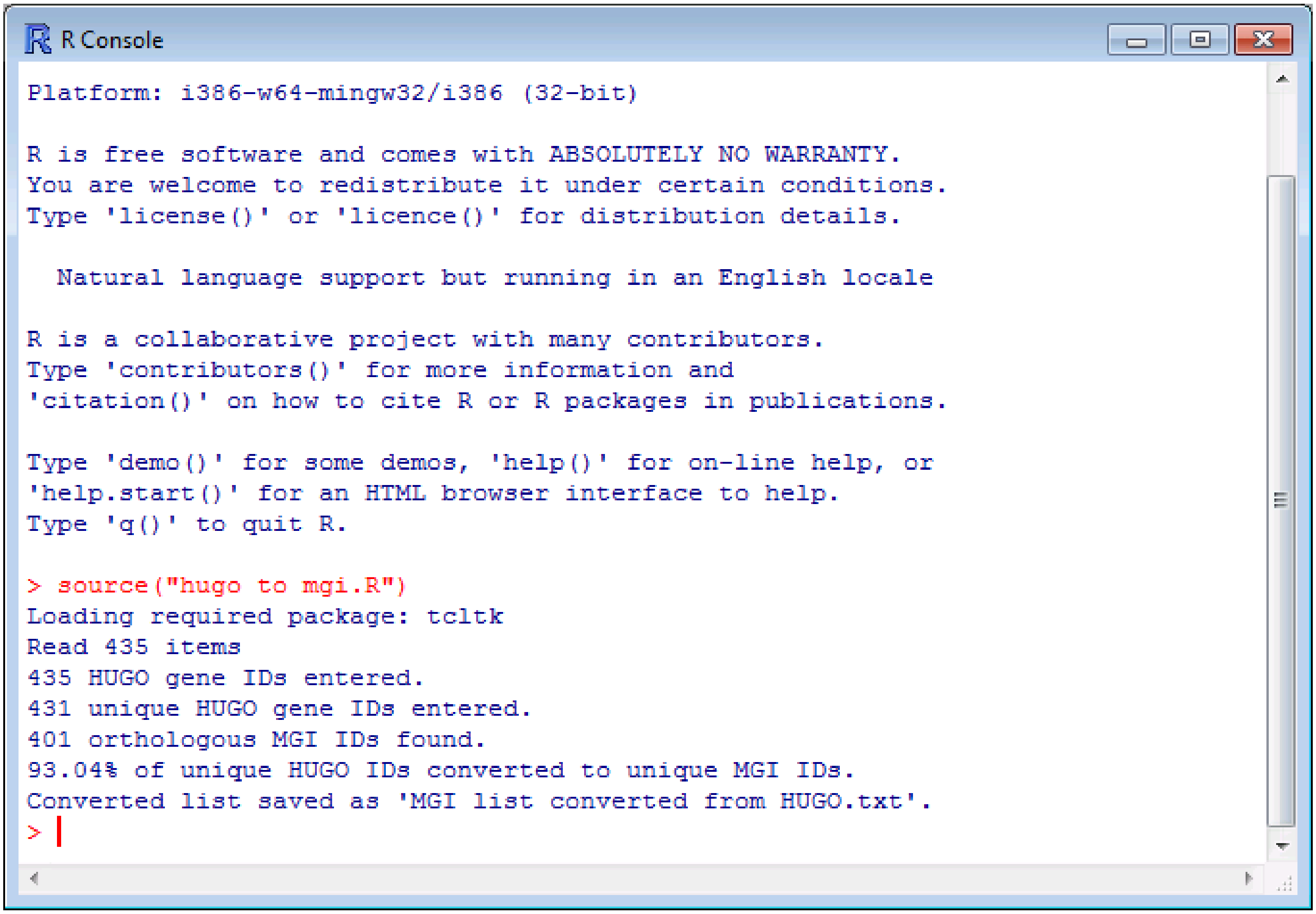

In this demonstration, the HUGO to MGI conversion tool successfully converted ~93% of HUGO genes into MGI format.

After a successful conversion, a new text file is written by the program into the MSET folder, called "MGI list converted from HUGO.txt". The user will likely want to rename this file manually to give it a more descriptive title.

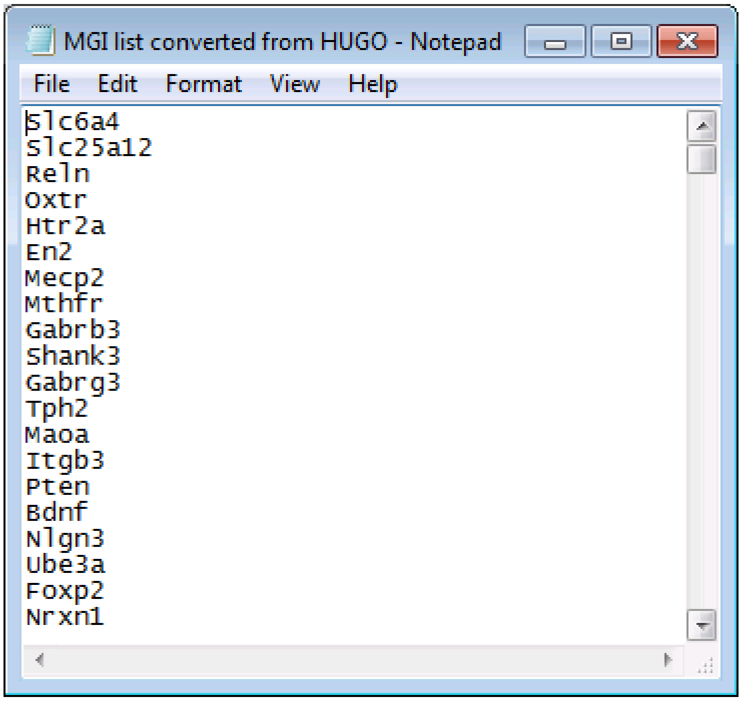

The converted gene list now contains a single column of MGI gene IDs.

Expression results should be formatted the same way as the disease associated gene list – as a single column text file of gene IDs with no header. After conducting a microarray experiment, the user will generally have a large results file with a variety of detailed information. It is essential to order results by significance, with the most significant gene IDs at the top, and the least significant at the bottom. Then, remove all information except for the ordered gene IDs and save as a text file.

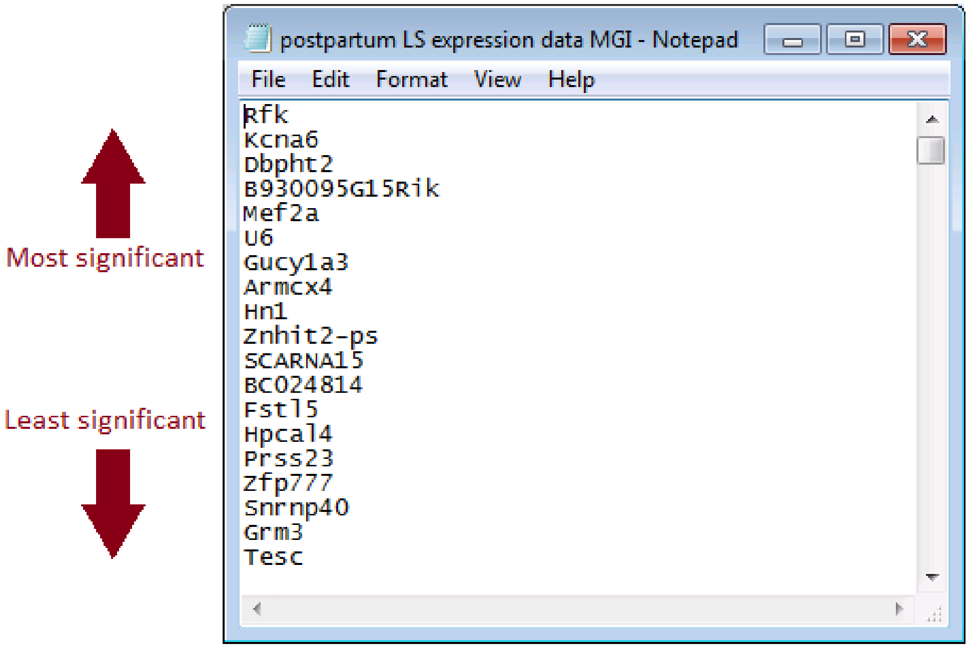

Postpartum mouse LS expression results as a column of MGI gene symbols arranged by significance.

To summarize, all input files must adhere to the following format rules:

1. Text file (.txt)
2. Single column of gene IDs
3. No column header
4. Expression results ordered by significance
5. Consistent gene ID nomenclature between files

Once the files are prepared, MSET can then analyze enrichment of disease associated gene lists within the expression results.

IV. Conducting MSET analysis

To use MSET, R and the MSET folder must both be downloaded. After R has been installed, launch R from its installation location on the hard drive.

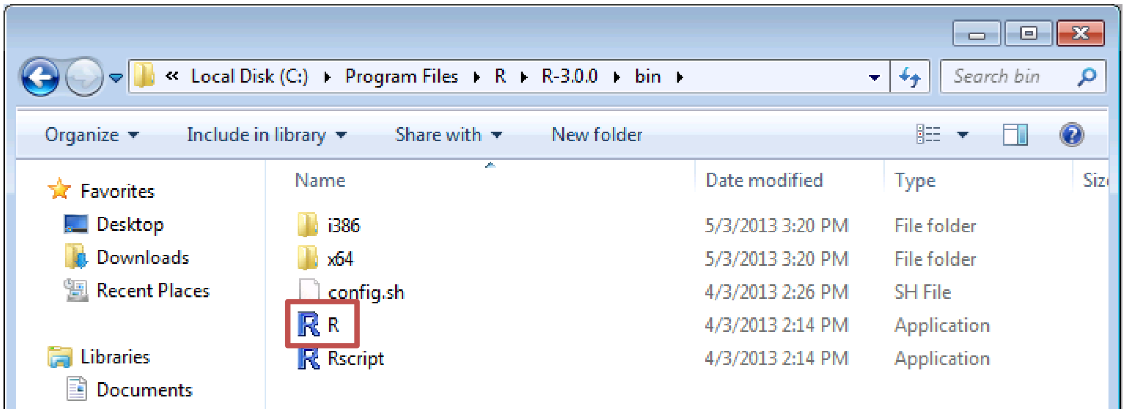

Launch R by executing the application from its installation location (red box).

Next, the MSET folder must be selected as R's working directory.

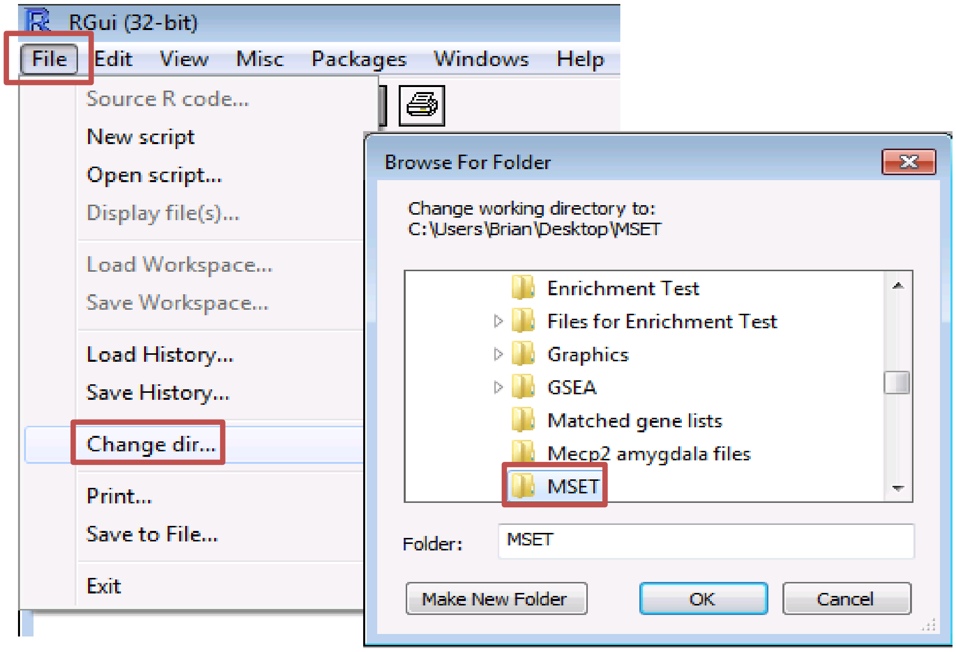

To designate the MSET folder as the working directory, click "File", "Change dir...", and select the MSET folder from its location on the hard drive. These steps are highlighted by red boxes.

After the MSET folder has been set as R's working directory, MSET can be launched by typing `source("mset.R")` in the R command console and pressing ENTER.

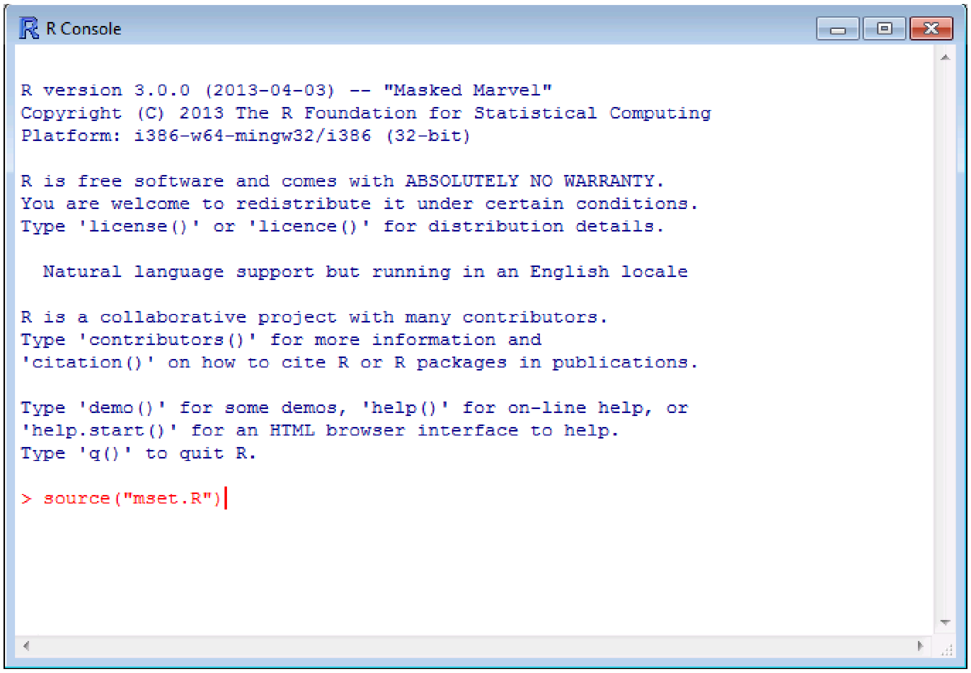

Launch MSET in the R console with the appropriate command line.

When prompted, select the expression data within which enrichment of disorder/disease linked genes will be assessed.

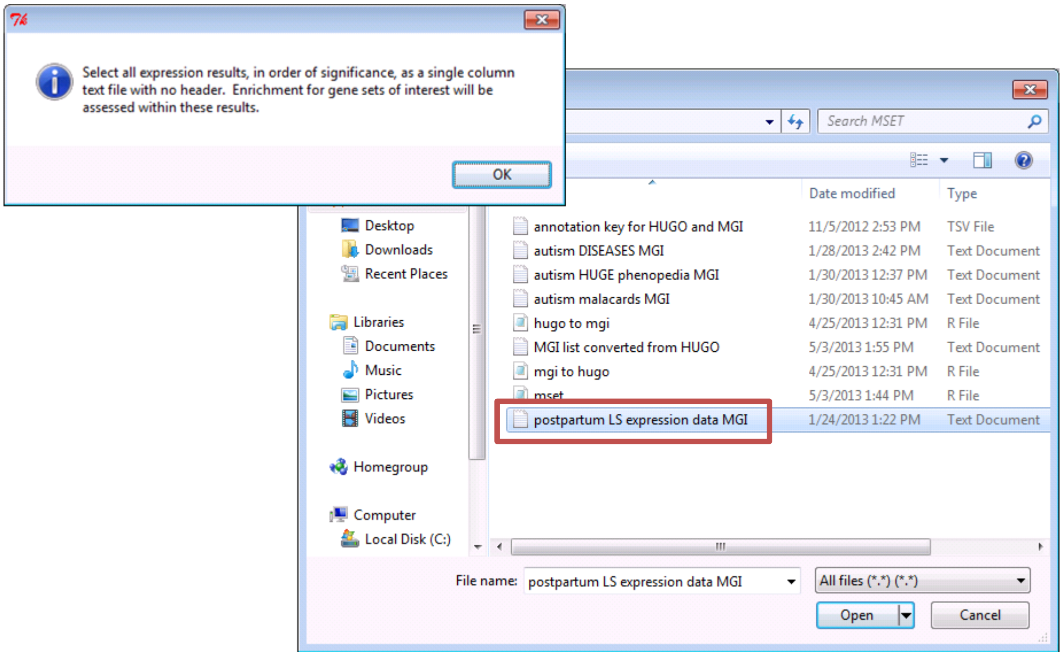

Select only one file of expression data. A sample set of postpartum mouse LS data is included in the MSET folder (red box).

Next, a prompt will ask you to select one or more databases of genes of interest. These can be lists of genes associated with any disease or biological phenomenon of interest. There is no defined maximum number of lists for which enrichment can be assessed in a single series of MSET analyses, but the default graphical output window can only accommodate 16 graphs at once – more than that will cause an error message and program failure. However, the graphical output window can be manually expanded to fit more graphs.

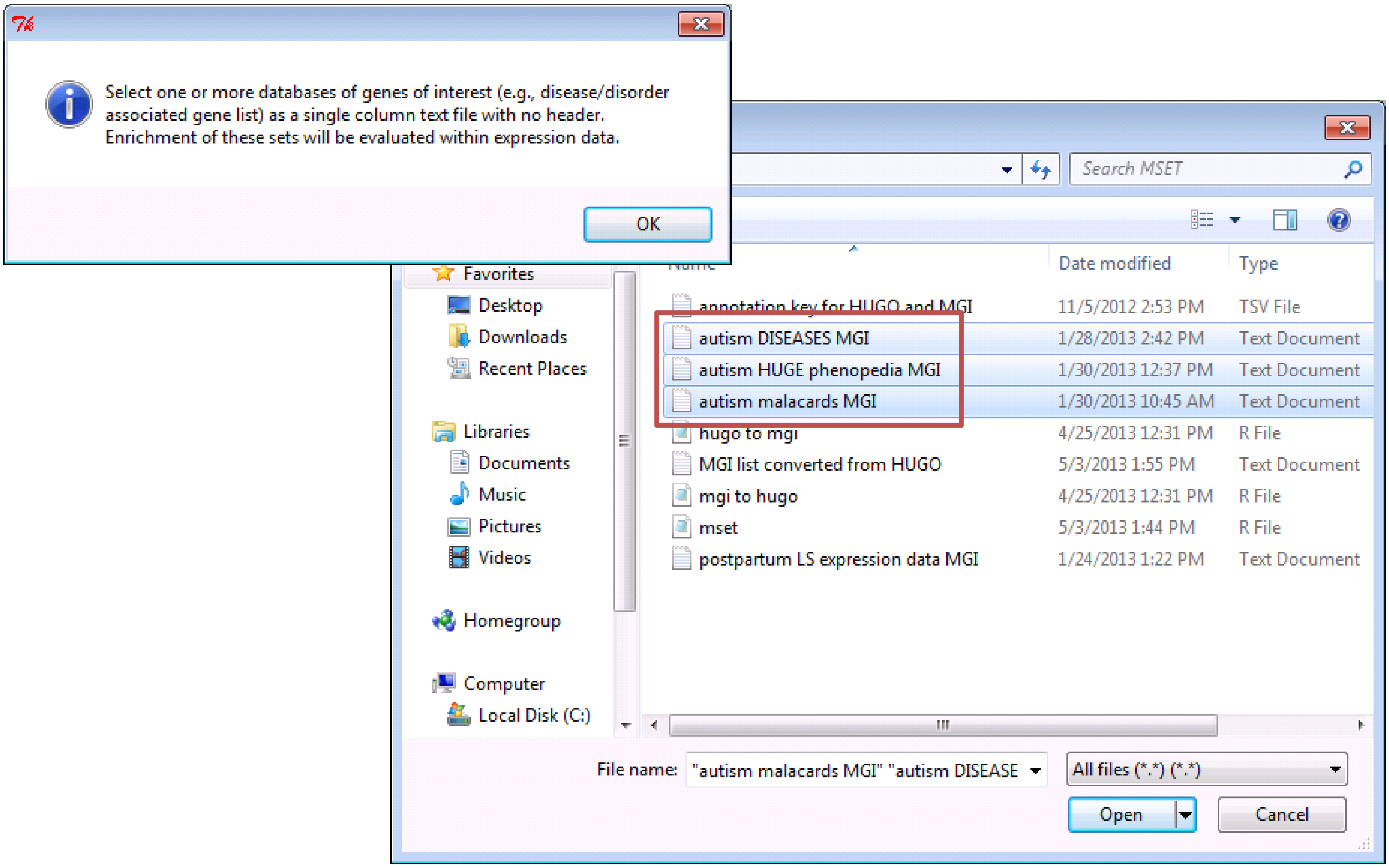

Select gene lists of interest. Enrichment of these gene lists will be assessed within expression results. In this demonstration, three disease gene databases have been selected.

Now, the MSET program will ask you to specify two parameters via text prompts. First, you must select how many of the most significant expression results in which you would like to assess enrichment. Because genes in the expression results file are ordered by significance, entering "1000" would tell MSET to use the top 1000 genes from that list. To use a particular p-value as a significance threshold, the user needs to examine their raw microarray results (including p-values and other data) to find the number of genes that correspond to that particular cutoff. In our postpartum mouse LS results, 1002 gene changes had an FDR-adjusted p-value less than 0.25.

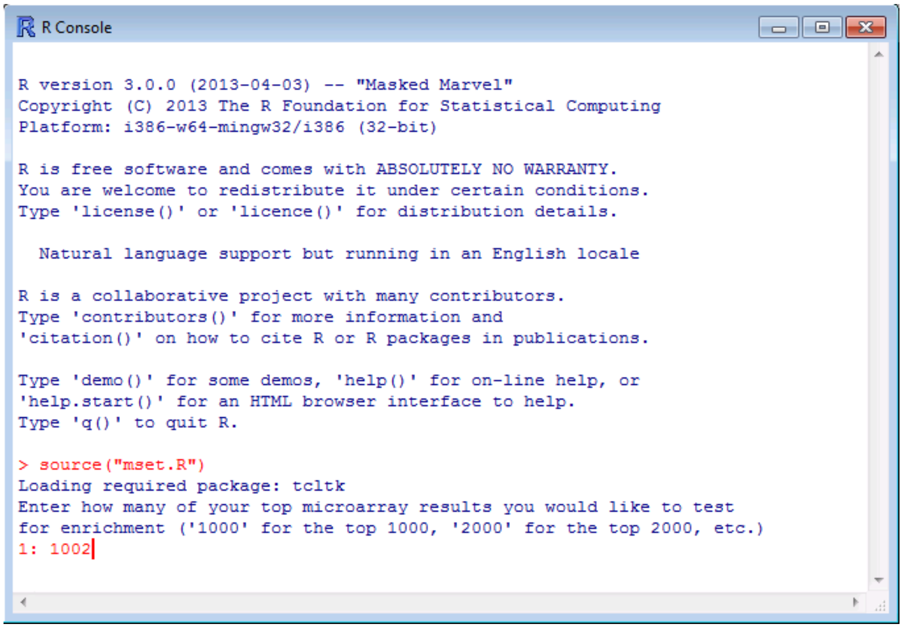

Choose how many of your top expression results you would like to test for enrichment. In our demonstration analysis, we tested the top 1002 genes.

The second text prompt asks how many simulations to generate in each analysis. As a randomization test, the accuracy of the test increases with the number of randomizations. For our demonstration analysis, we generate 10,000 simulated results by sampling randomly and without replacement from the entirety of the expression results, also known as the microarray background.

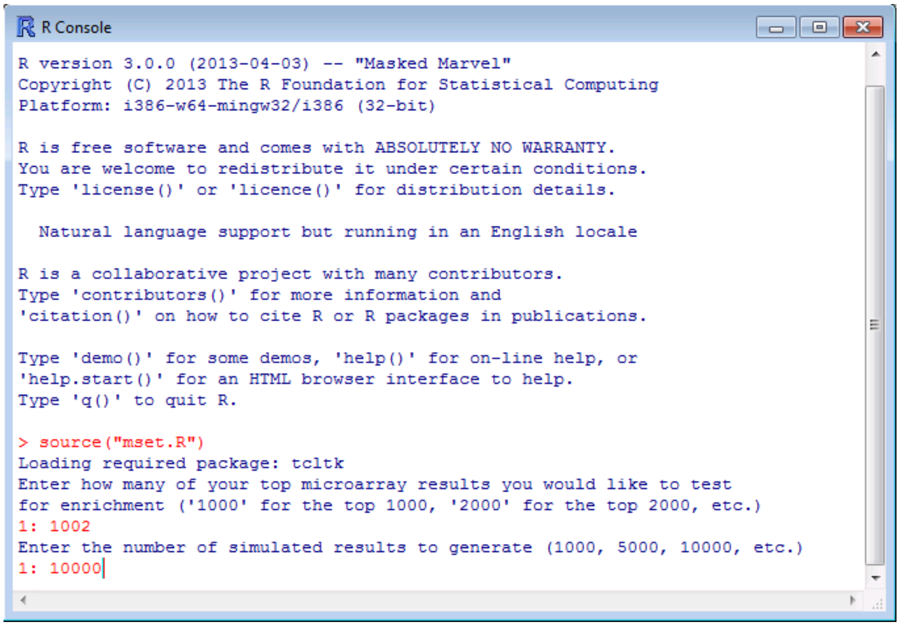

Enter the number of simulated results for MSET to generate in a single analysis. The distribution of matches to database in simulated results is used to derive a p-value for the actual significant results.

For each combination of disease associated gene lists entered and the expression results selected, enrichment analysis is performed.

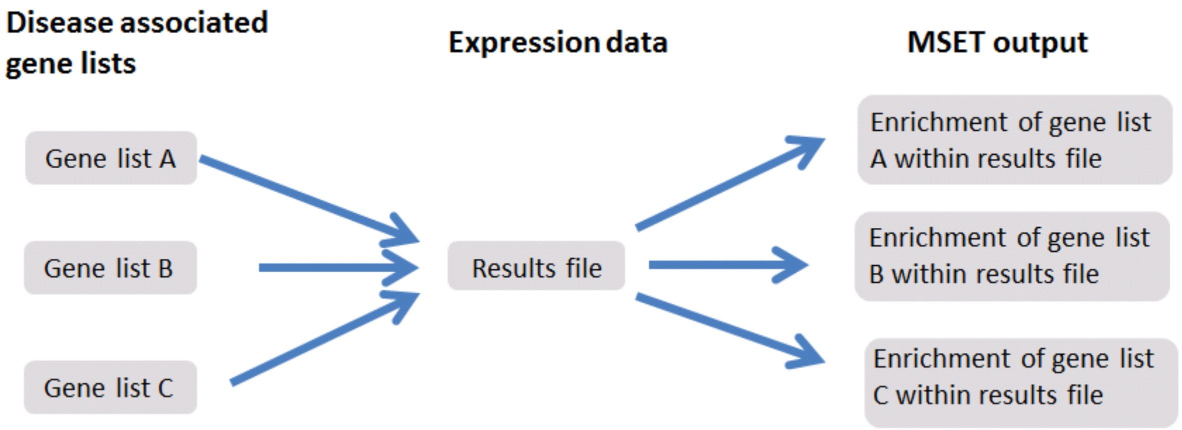

The modular nature of MSET – enrichment for numerous gene lists associated with a disease of interest can be simultaneously evaluated in a set of microarray expression results.

For each analysis, a text readout and graphical output are produced. The text readout is a summary with multiple sections. The first section, INPUT FILES, summarizes which files were selected as input and how many gene IDs were found in each.

The RESULTS section shows how many simulated results were generated of a length that matches the actual number of significant expression results. It shows how many matches to database were found in the actual results, and how many simulated results had at least as many matches to database as were observed in the true results. A p-value is derived from these observations.

The ADDITIONAL INFO section describes what percent of significant expression results are matches to the disease linked gene database, and what percent of the entire microarray background are matches to the database. Fold enrichment is calculated simply as the ratio of these values

The MATCHES TO DATABASE section individually names genes in the significant expression results which also appear in the disease associated gene database.

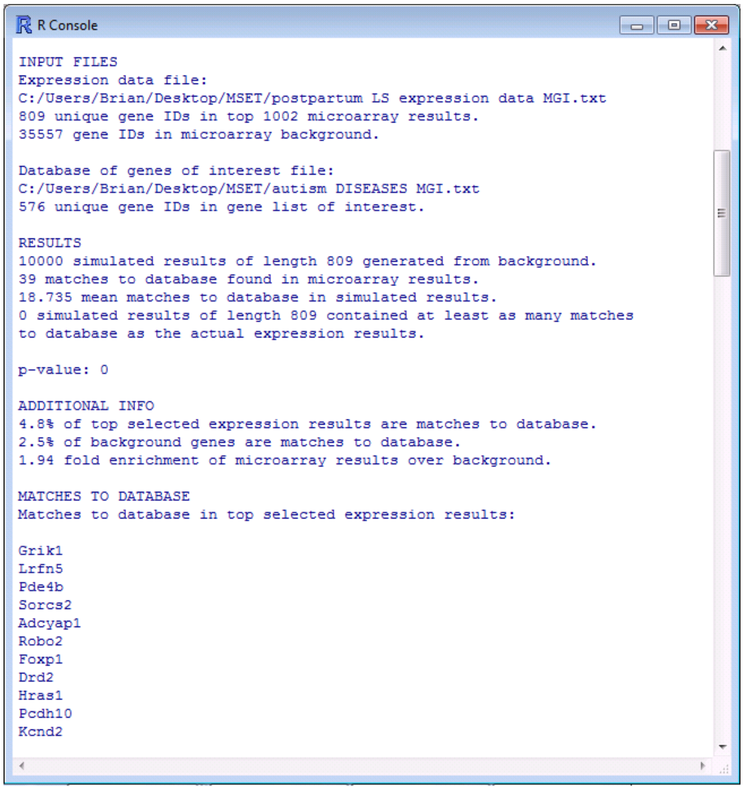

```
R Console

INPUT FILES
Expression data file:
C:/Users/Brian/Desktop/MSET/postpartum LS expression data MGI.txt
809 unique gene IDs in top 1002 microarray results.
35557 gene IDs in microarray background.

Database of genes of interest file:
C:/Users/Brian/Desktop/MSET/autism DISEASES MGI.txt
576 unique gene IDs in gene list of interest.

RESULTS
10000 simulated results of length 809 generated from background.
39 matches to database found in microarray results.
18.735 mean matches to database in simulated results.
0 simulated results of length 809 contained at least as many matches
to database as the actual expression results.

p-value: 0

ADDITIONAL INFO
4.8% of top selected expression results are matches to database.
2.5% of background genes are matches to database.
1.94 fold enrichment of microarray results over background.

MATCHES TO DATABASE
Matches to database in top selected expression results:

Grik1
Lrfn5
Pde4b
Sorcs2
Adcyap1
Robo2
Foxp1
Drd2
Hras1
Pcdh10
Kcnd2
```

The text readout of a single MSET analysis.

In addition to the text readout, MSET results are also shown in a graphical format. A graph window is produced when MSET runs, and is populated with graphs as each analysis is completed. In our demonstration, three graphs are produced. Each graph is a density curve showing how many randomly generated simulated results had a particular number of matches to the disease associated gene database. A vertical blue line is placed on this graph to show where the number of matches found in the true expression results fall with respect to this distribution. A line far to the right tail of the curve reflects a very low probability that the number of disease associated genes detected in the true results occurred by chance. For reference, the database used in each analysis is given as a truncated file name underneath its respective graph.

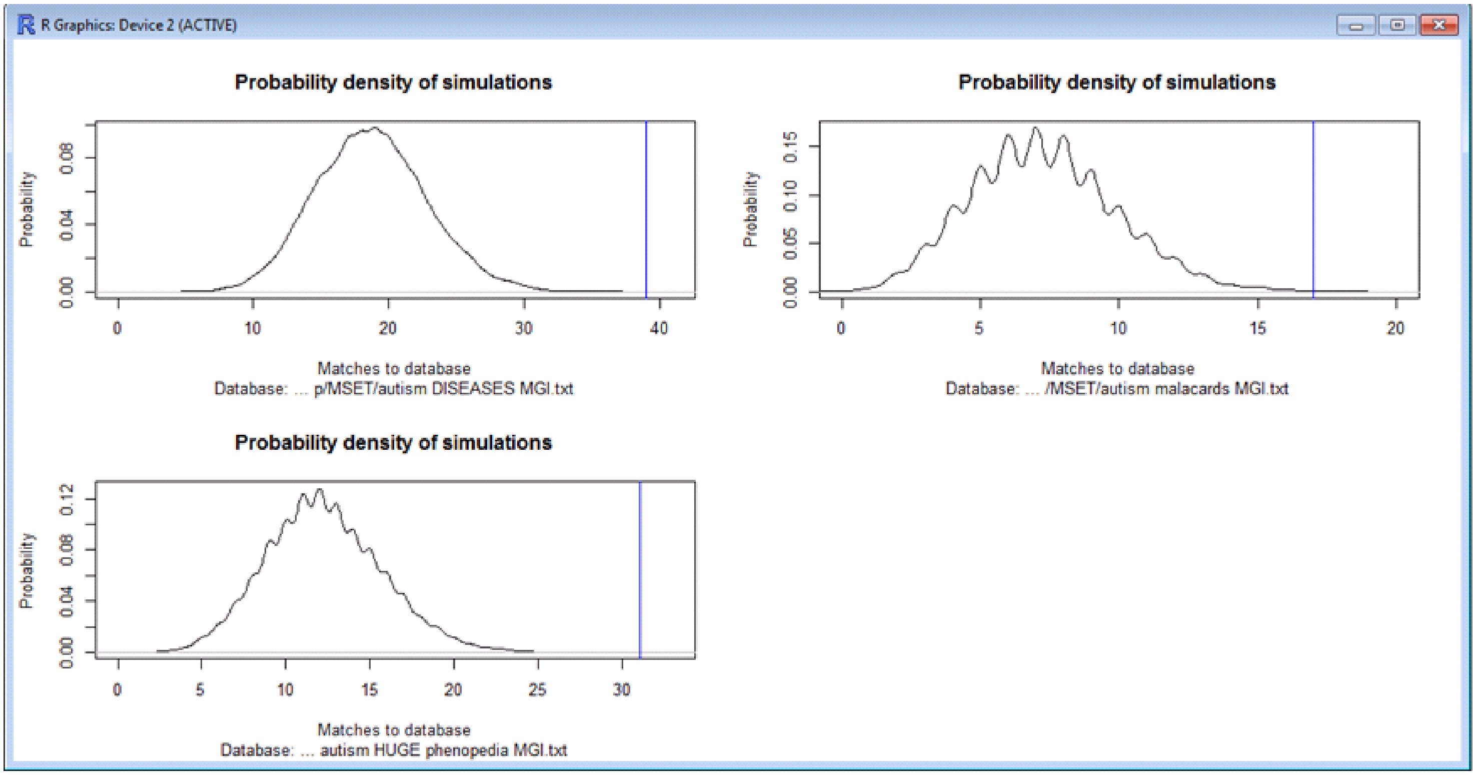

The graphical MSET output pane. All analyses are shown at once.
